# Supplementary figures and images for: Proteomic analysis identifies novel binding partners of BAP1
Source: PLoS One. 2021 Sep 30;16(9):e0257688. doi: 10.1371/journal.pone.0257688 (PMC8483321; doi:10.1371/journal.pone.0257688)

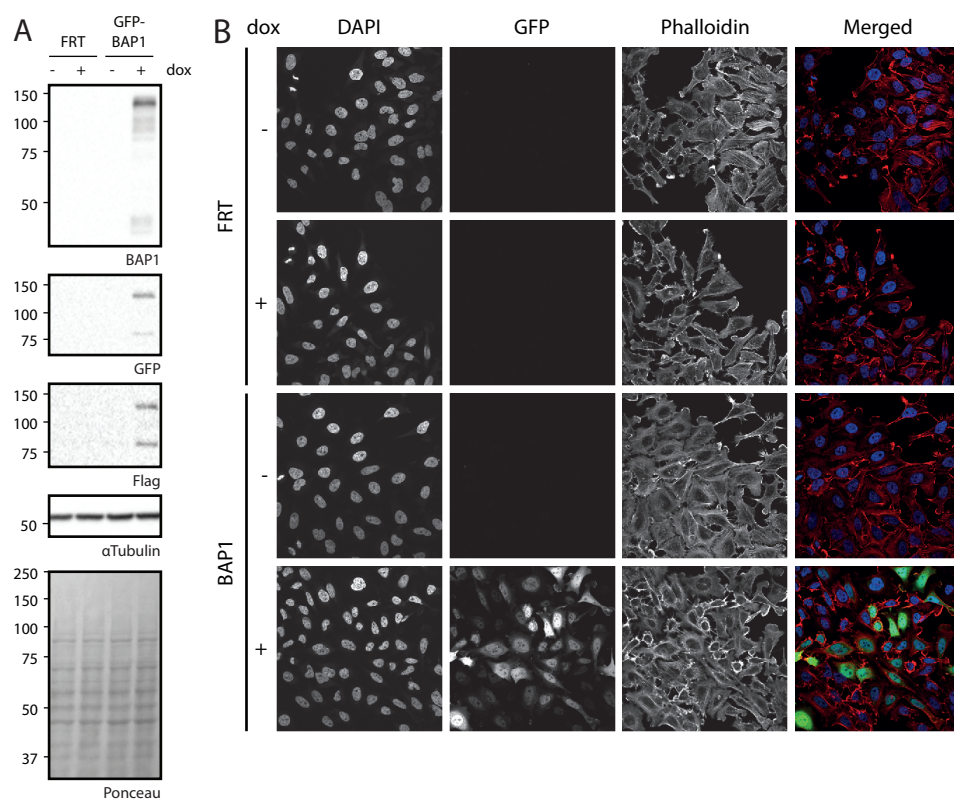

Supplement: S1 Fig — (A). Immunoblot analysis of GFP-BAP1 in HeLa FRT cells upon dox induction. Lysates of FRT parental or GFP-BAP1 expressing cells was analyzed on blot using listed antibodies. (B). Confocal microscopy for cell lines used in (A) shows GFP-BAP1 expression and localization in cells. Cells were grown on coverslips and stained using DAPI and Phalloidin-633 and mounted on microscopy slides. (PDF) [file pone.0257688.s001.pdf]

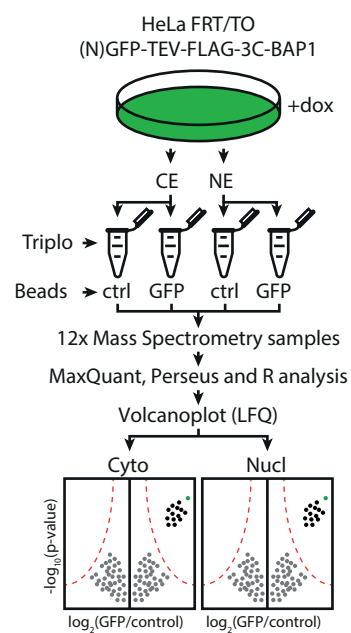

Supplement: S2 Fig — (PDF) [file pone.0257688.s002.pdf]

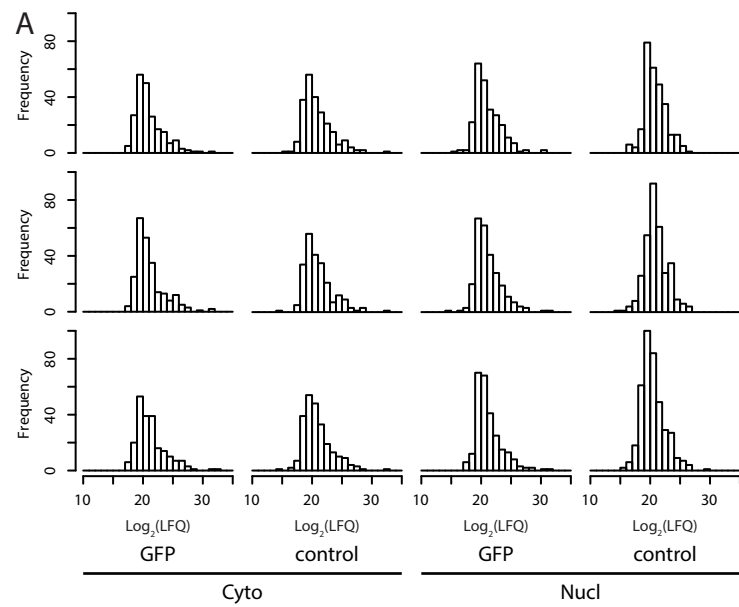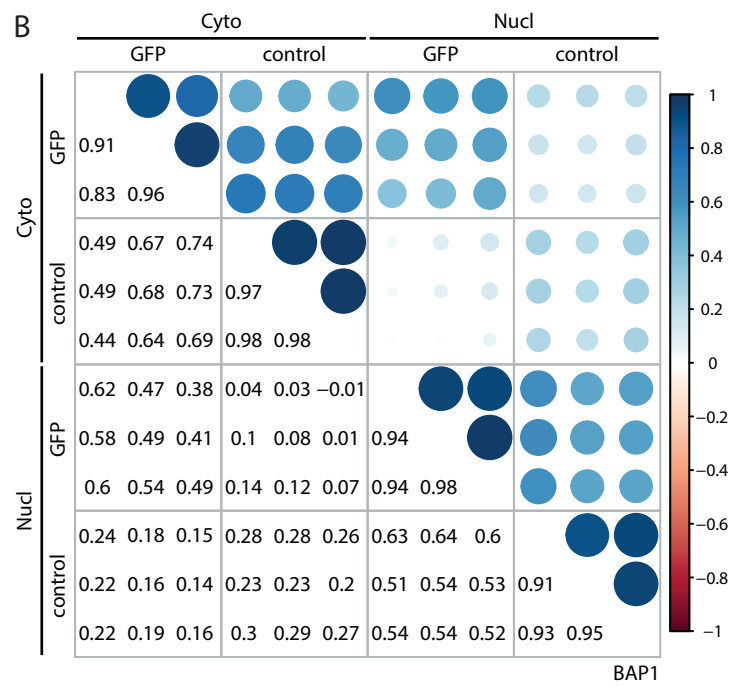

Supplement: S3 Fig — (A). Histograms of individual mass spectrometry samples. (B). Correlation plots of samples analyzed in (A). Correlation coefficients between log2(LFQ) values of all individual samples within cell lines are depicted as a number (lower triangle) or visually as colored circle (upper triangle). (PDF) [file pone.0257688.s003.pdf]

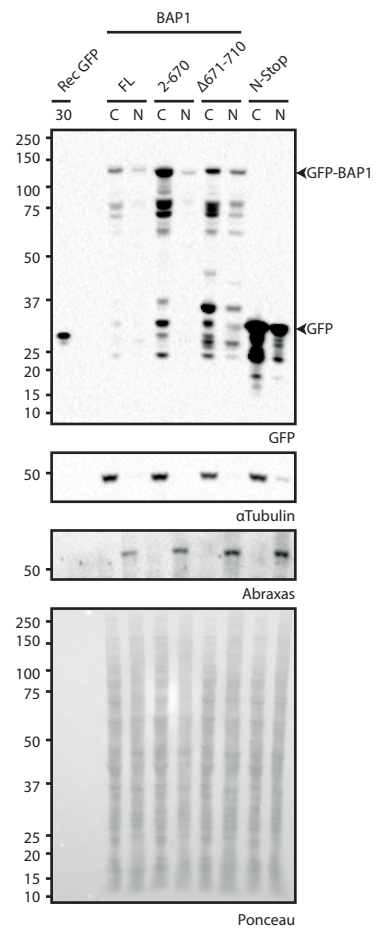

Supplement: S5 Fig — Equal amount of extracts used for mass spectrometry analysis in Fig 4 were blotted and analyzed using the listed antibodies. Tubulin is used as a cytoplasmic protein marker and Abraxas is used as a nuclear protein marker. (PDF) [file pone.0257688.s005.pdf]

A

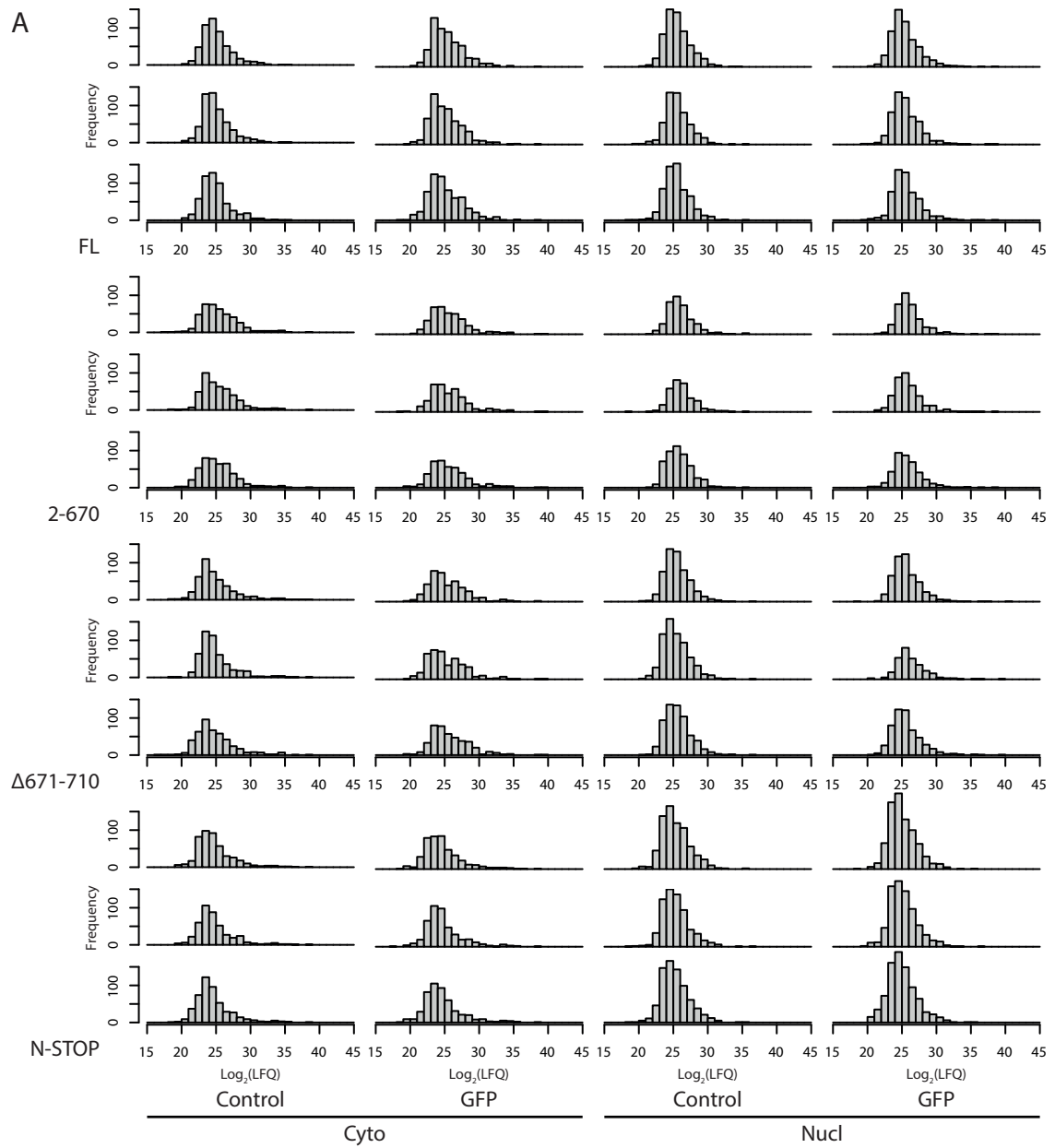

B

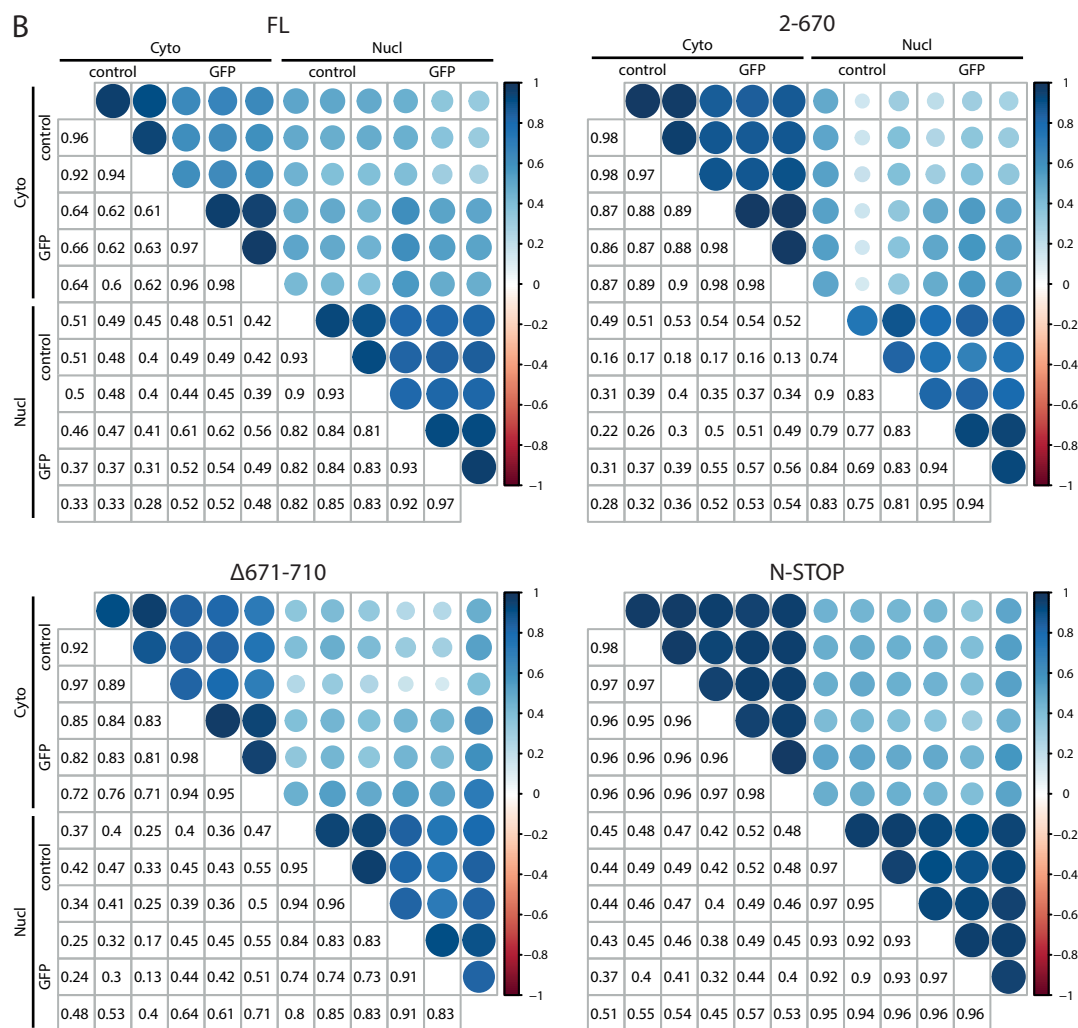

Supplement: S6 Fig — (A). Histograms of individual mass spectrometry samples. (B).–Correlation plots of samples analyzed in (A). Correlation coefficients between log2(LFQ) values of all individual samples within cell lines are depicted as a number (lower triangles) or visually as colored circle (upper triangles). (PDF) [file pone.0257688.s006.pdf]
