# Supplementary material for: Proteomic analysis identifies novel binding partners of BAP1
Source: PLoS One. 2021 Sep 30;16(9):e0257688. doi: 10.1371/journal.pone.0257688 (PMC8483321; doi:10.1371/journal.pone.0257688)
Supplement: S4 Fig — (A and C). TIDE analysis for CRISPR clones 4C5 and 4D2 respectively. (A). The TIDE algorithm was unable to identify the deletions in the 4C5 clone within its search window of -50 to +50 bp. (C). Clone 4D2 was found to contain a 1 and 5bp deletion. (B and D) CRISP-ID analysis for CRISPR clones 4C5 and 4D2 respectively. Top sequence is the used reference sequence. Lower 2 sequences contain the deconvoluted CRISPR clone sequencing results. Colors represent full sequence alignment between reference sequence and deconvoluted sequences. (B). CRISP-ID shows clone 4C5 to contain a 35 and 88bp deletion. (D). Clone 4D2 contains a 1 and 5bp deletion as observed in (C). (PDF) [file pone.0257688.s004.pdf]

A

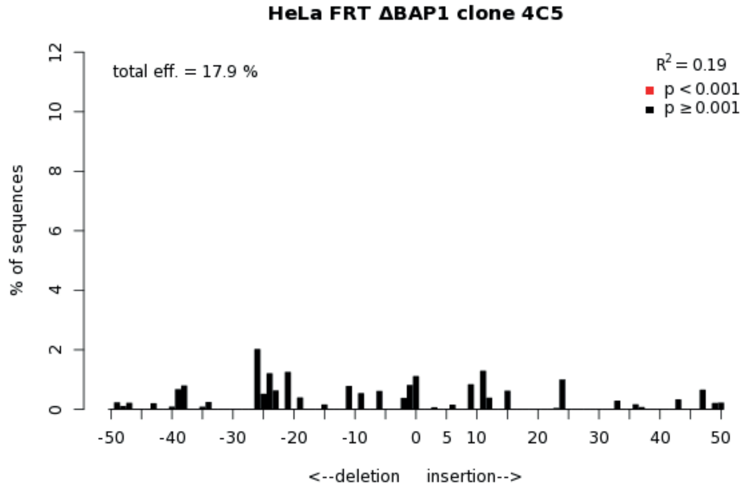

B HeLa FRT ΔBAP1 clone 4C5

```
                       1                                                                                                100
Reference sequence   1 ACACCTGAGTGATGACGCAGTGCAAAGGATTAATGGGAGAAAGAAGGGAATGCTGATTGTCTTCTCCCCTTTGGCTGATCTGGCTCTGCCCCTTACATCC 100
               Δ88   1 ACACCTGAGTGATGACGCAGTGCAAAGGATTAATGGGAGAAAGAAGGGAATGCTGATTGTCTTCTCCCCTTTGGCTGATCTGGCTCTGCCCCTTACATCC 100
               Δ35   1 ACACCTGAGTGATGACGCAGTGCAAAGGATTAATGGGAGAAAGAAGGGAATGCTGATTGTCTTCTCCCCTTTGGCTGATCTGGCTCTGCCCCTTACTTCC 100

                       101                                                                                              200
Reference sequence 101 CCCAGCCCTGTATATGGATTTATCTTCCTGTTCAAATGGATCGAAGAGCGCCGGTCCCGGCGAAAGGTCTCTACCTTGGTGGATGATACGTCCGTGATTG 200
               Δ88 101 CCCAGCCCTGTATATGGATTTATCTTCCTGTTCAAATGGATCGAAGAGCGCCGGTCCCGTCGAAAGGTCTCT----------------------------- 172
               Δ35 101 CCAAGCCCTGTATATGGATTTATCTTCCTGTTCAAATGGATCGAAGAGCGCCGTGCC-----------------------GGA------------GATTG 165

                       201                                                                                            299
Reference sequence 201 ATGATGATATTG-TGAATAACATGTTCTTTGCCCACCAGGTCTGCTGGACTCTGTGCTTTGTTTGGAGGGTGGGATGCTGCCATGTTTTTGCTTGGGA 297
               Δ88 172 -------------------------------------------------------------ATTGGTAGTGGGGATACT 189
               Δ35 166 AGGACGACATTGATGA 181
```

C

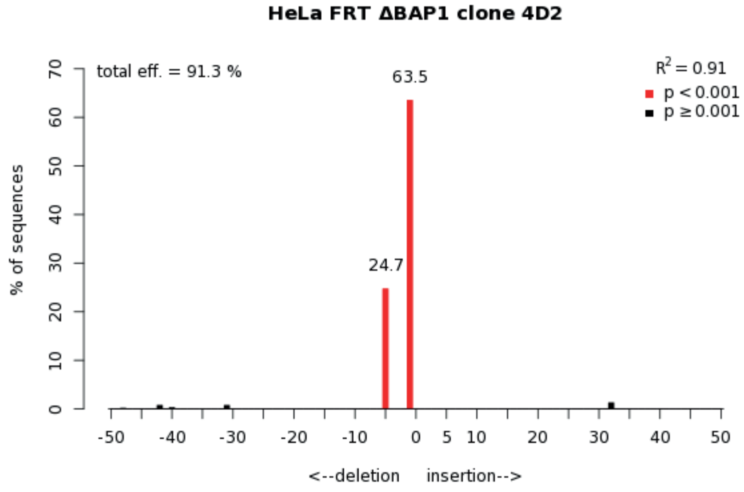

D HeLa FRT ΔBAP1 clone 4D2

```
                       1                                                                                                100
Reference sequence   1 CCTGTTCAAATGGATCGAAGAGCGCCGGTCCCGGCGAAAGGTCTCTACCTTGGTGGATGATACGTCCGTGATTGATGATGATATTGTGAATAACATGTTC 100
                Δ1   1 CCTGTTCAAATGGATCGAAGAGCGCCGGTCCCGGCGAAAGGTCTCT-CCTTGGTGGATGATACGTCCGTGATTGATGATGATATTGTGAATAACATGTTC 99
                Δ5   1 CCTGTTCAAATGGATCGAAGAGCGCCGGTCCCGGCGAAAGGTCTCTA-----GTGGATGATACGTACGTGATTGATGATGATATTGTGAATAACATGTTC 95
```
