# Supplementary material for: Proteomic analysis identifies novel binding partners of BAP1
Source: PLoS One. 2021 Sep 30;16(9):e0257688. doi: 10.1371/journal.pone.0257688 (PMC8483321; doi:10.1371/journal.pone.0257688)
Supplement: S1 File — (DOCX) [file pone.0257688.s007.docx]

**Supplementary results**

***GFP-BAP1 cell line analysis***

BAP1 immunoblot analysis of wild-type and GFP- BAP1 full-length cells shows a band at the expected size (linker (31 kDa) + BAP1 (80 kDa) = 111 kDa, Supplemental Figure 1A). Some smaller products are visible below the prominent band, suggesting partial degradation of the fusion construct. GFP and FLAG immunoblots show the same band at the expected height and a smaller distinct band below. This suggests the degradation of the BAP1 fusion construct is probably BAP1 related and not due to the degradation of GFP-FLAG. Possibly this degradation is related to biological regulation of BAP1. Next, confocal microscopy analysis of GFP-BAP1 was done to investigate fusion construct localization. GFP-BAP1 shows a preference for localization in the nucleus versus the cytoplasm, as is typically observed for BAP1 (Supplementary Figure 1B,[1]).

***TIDE and CRISP-ID analysis of HeLa FRT ΔBAP1 cells***

TIDE and CRISP-ID analysis showed a genomic deletion of 35 and 88 bp for clone 4C5 and a 1 and 5 bp deletion for clone 4D2, rendering BAP1 out-of-frame in both clones. Because selection for CRISPR clones and FRT mediated stable cell line generation both use the puromycin selection marker it is important to check the cell lines for puromycin sensitivity. Both 4C5 and 4D2 clones are still sensitive to puromycin and thus usable for FRT mediated stable cell line generation (Figure 2B).

**Supplementary figure legends**

Supplemental Figure 1 – GFP-BAP1 expression and localization. (A). Immunoblot analysis of GFP-BAP1 in HeLa FRT cells upon dox induction. (B). Confocal microscopy shows GFP-BAP1 expression and localization in cells.

Supplemental Figure 2 – AP-MS workflow for GFP-tagged proteins.

Supplemental Figure 3 – Quality control of AP-MS experiment belonging to Figures 1A and 1B. (A). Histograms of individual mass spectrometry samples. (B). Correlation plots of samples analyzed in (A). Correlation coefficients between log2(LFQ) values of all individual samples within cell lines are depicted as a number (lower triangle) or visually as colored circle (upper triangle).

Supplemental Figure 4 – CRISPR clone genotype analysis. (A and C). TIDE analysis for CRISPR clones 4C5 and 4D2 respectively. (A). The TIDE algorithm was unable to identify the deletions in the 4C5 clone within its search window of -50 to +50 bp. (C). Clone 4D2 was found to contain a 1 and 5bp deletion. (B and D) CRISP-ID analysis for CRISPR clones 4C5 and 4D2 respectively. Top sequence is the used reference sequence. Lower 2 sequences contain the deconvoluted CRISPR clone sequencing results. Colors represent full sequence alignment between reference sequence and deconvoluted sequences. (B). CRISP-ID shows clone 4C5 to contain a 35 and 88bp deletion. (D). Clone 4D2 contains a 1 and 5bp deletion as observed in (C).

Supplemental Figure 5 – Cytoplasmic and nuclear separation of BAP1 truncation mutant extracts belonging to Figure 4.

Supplemental Figure 6 – Quality control of AP-MS experiment belonging to Figure 4. – (A). Histograms of individual mass spectrometry samples. (B). – Correlation plots of samples analyzed in (A). Correlation coefficients between log2(LFQ) values of all individual samples within cell lines are depicted as a number (lower triangles) or visually as colored circle (upper triangles).

**Supplementary references**

1. Ventii, K.H., et al., *BRCA1-associated protein-1 is a tumor suppressor that requires deubiquitinating activity and nuclear localization.* Cancer Res, 2008. **68**(17): p. 6953-62.
