# Supplementary material for: Proteomic analysis identifies novel binding partners of BAP1
Source: PLoS One. 2021 Sep 30;16(9):e0257688. doi: 10.1371/journal.pone.0257688 (PMC8483321; doi:10.1371/journal.pone.0257688)
Supplement: S1 Raw images — (PDF) [file pone.0257688.s009.pdf]

Fig 1C

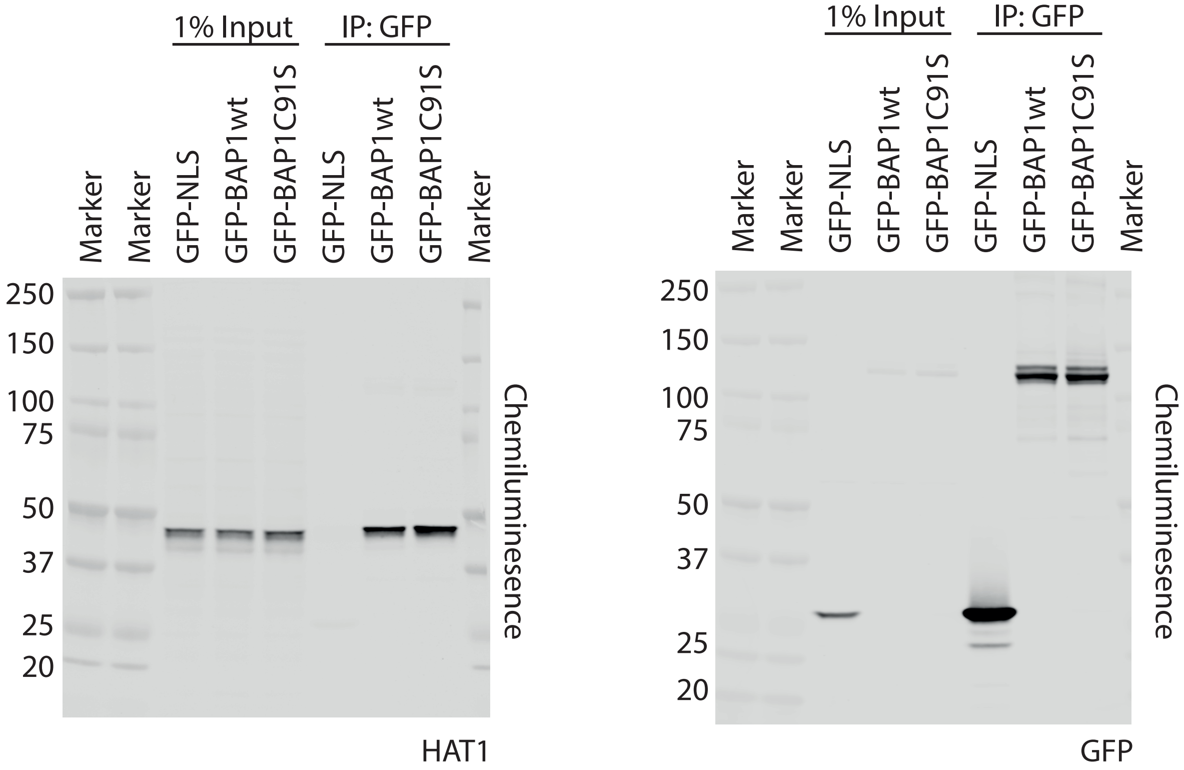
1% Input
IP: GFP
Marker
Marker
GFP-NLS
GFP-BAP1wt
GFP-BAP1C91S
GFP-NLS
GFP-BAP1wt
GFP-BAP1C91S
Marker
250
150
100
75
50
37
25
20
Chemiluminesence
HAT1
1% Input
IP: GFP
Marker
Marker
GFP-NLS
GFP-BAP1wt
GFP-BAP1C91S
GFP-NLS
GFP-BAP1wt
GFP-BAP1C91S
Marker
250
150
100
75
50
37
25
20
Chemiluminesence
GFP

Fig 1D

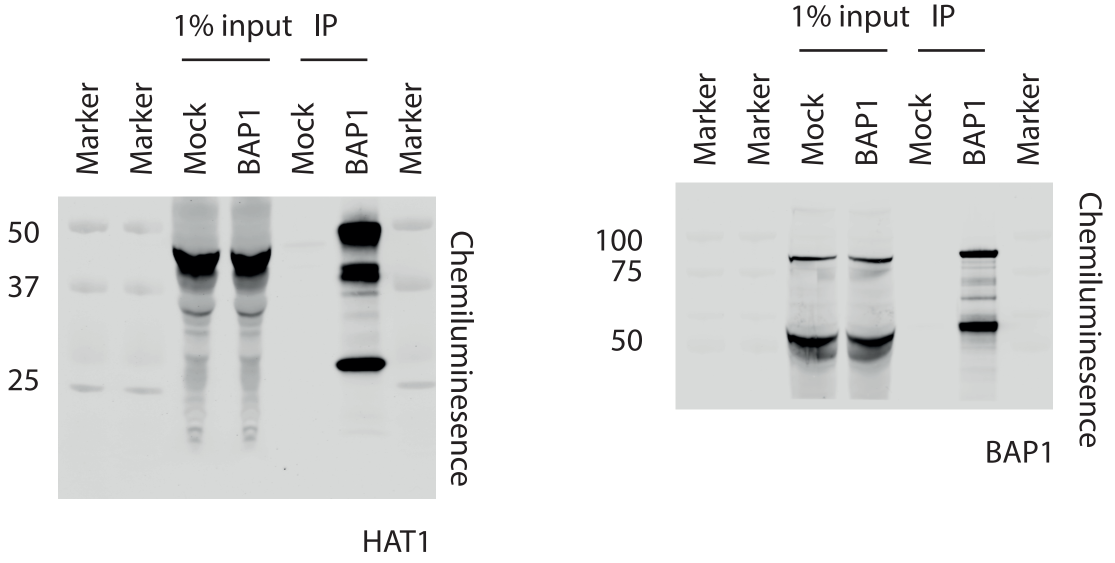
1% input
IP
Marker
Marker
Mock
BAP1
Mock
BAP1
Marker
50
37
25
Chemiluminesence
HAT1
1% input
IP
Marker
Marker
Mock
BAP1
Mock
BAP1
Marker
100
75
50
Chemiluminesence
BAP1

Fig 2A

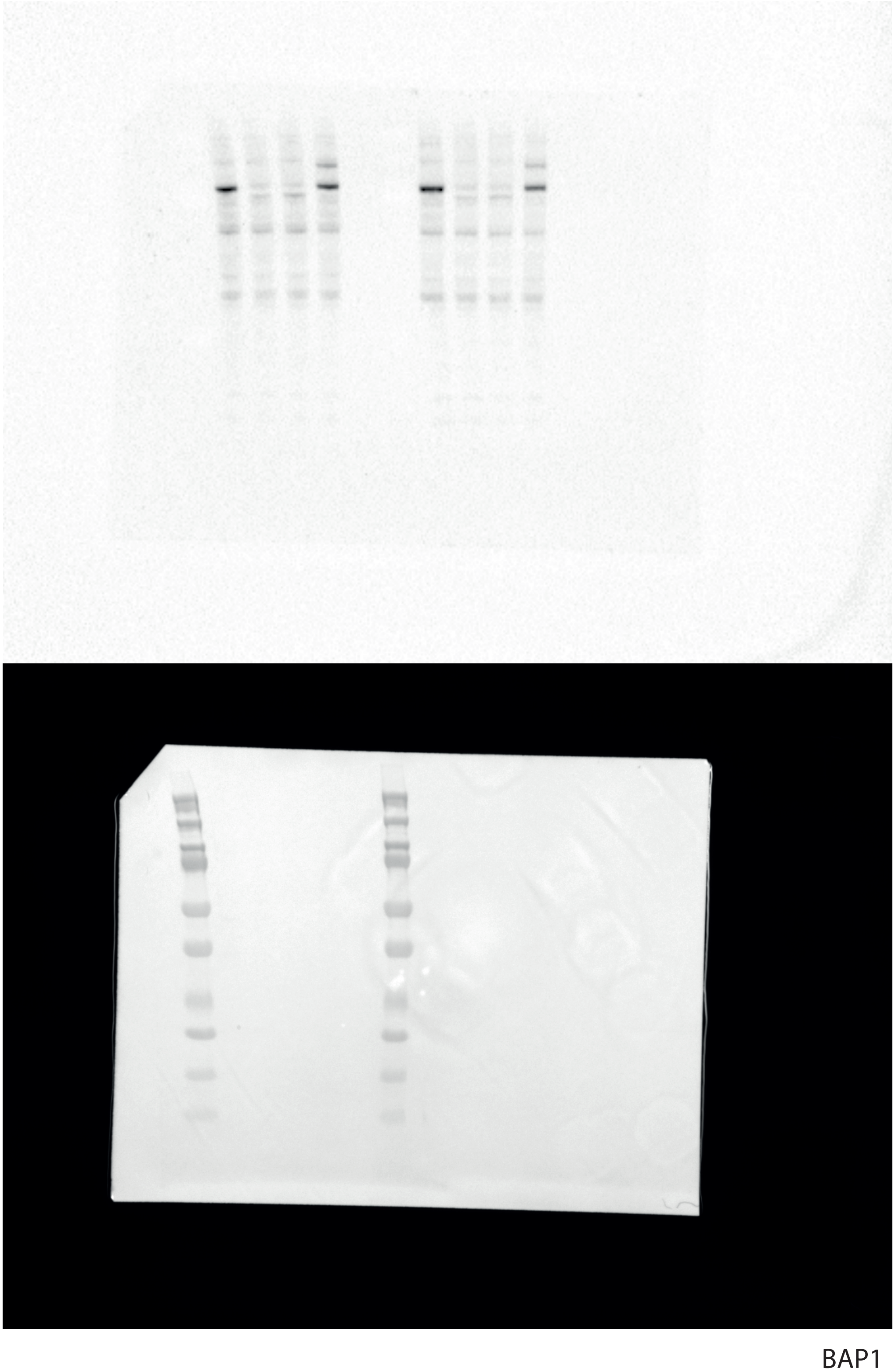

Marker
X
X
X
X
Marker
HeLa FRT wt
HeLa FRT ΔBAP1 #4C5
HeLa FRT ΔBAP1 #4D2
HeLa FRT GFP-BAP1 -dox
Chemiluminesence
250
150
100
75
50
37
25
20
15
10
Photo

BAP1

Fig 2A

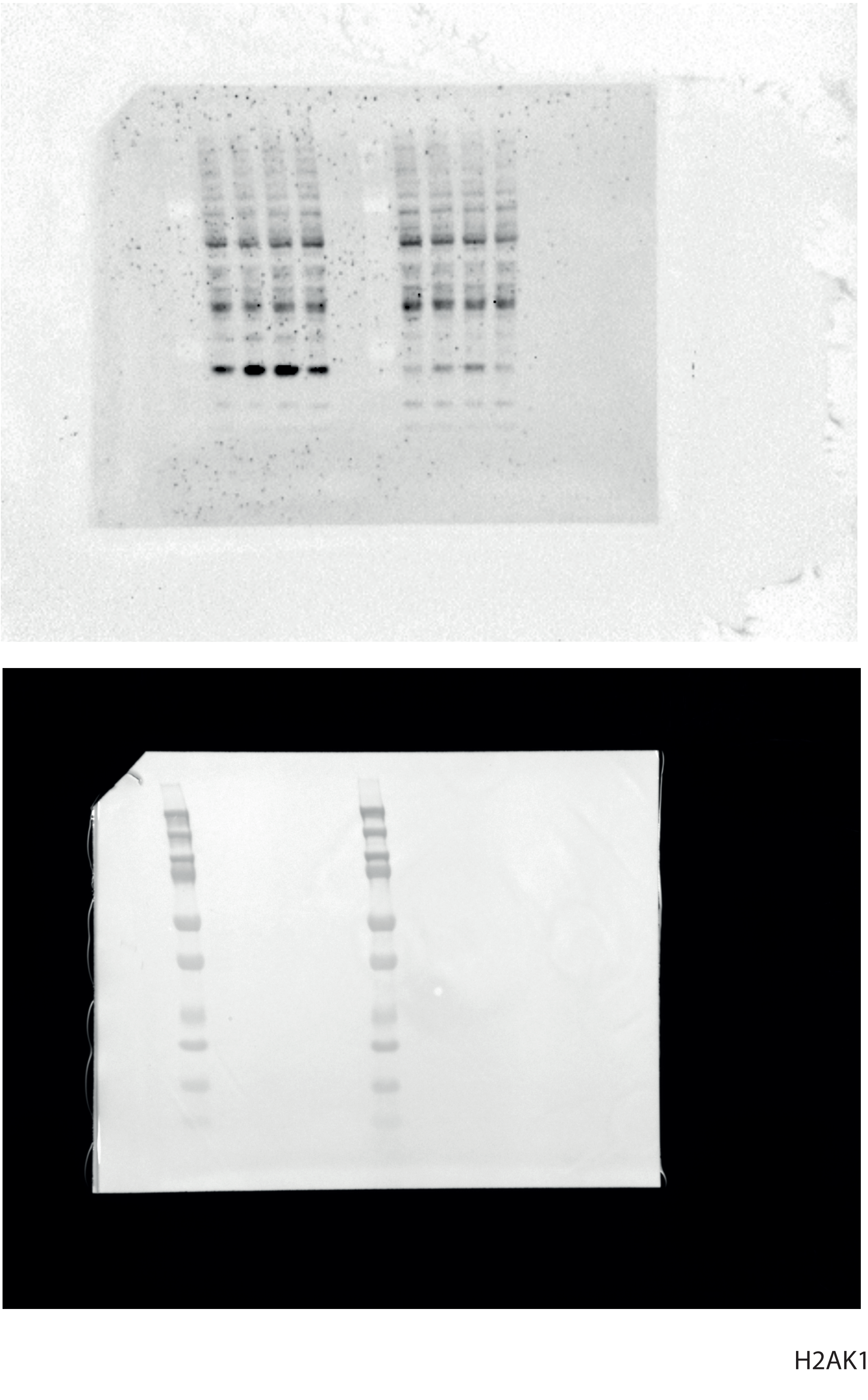

H2AK119ub

Fig 2A

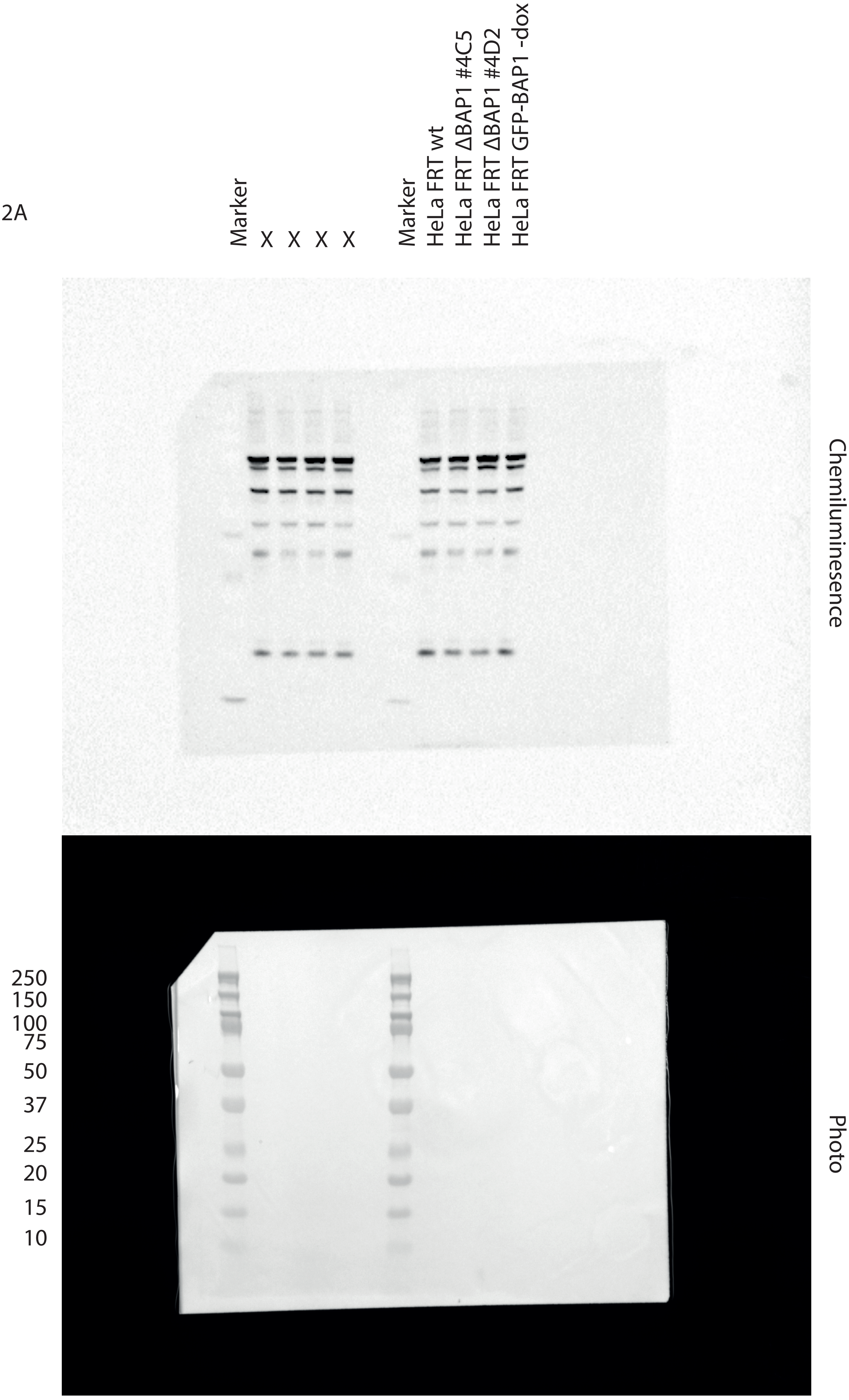
Marker
X
X
X
X
Marker
HeLa FRT wt
HeLa FRT ΔBAP1 #4C5
HeLa FRT ΔBAP1 #4D2
HeLa FRT GFP-BAP1 -dox
Chemiluminesence
250
150
100
75
50
37
25
20
15
10
Photo

H2A

Fig 2A

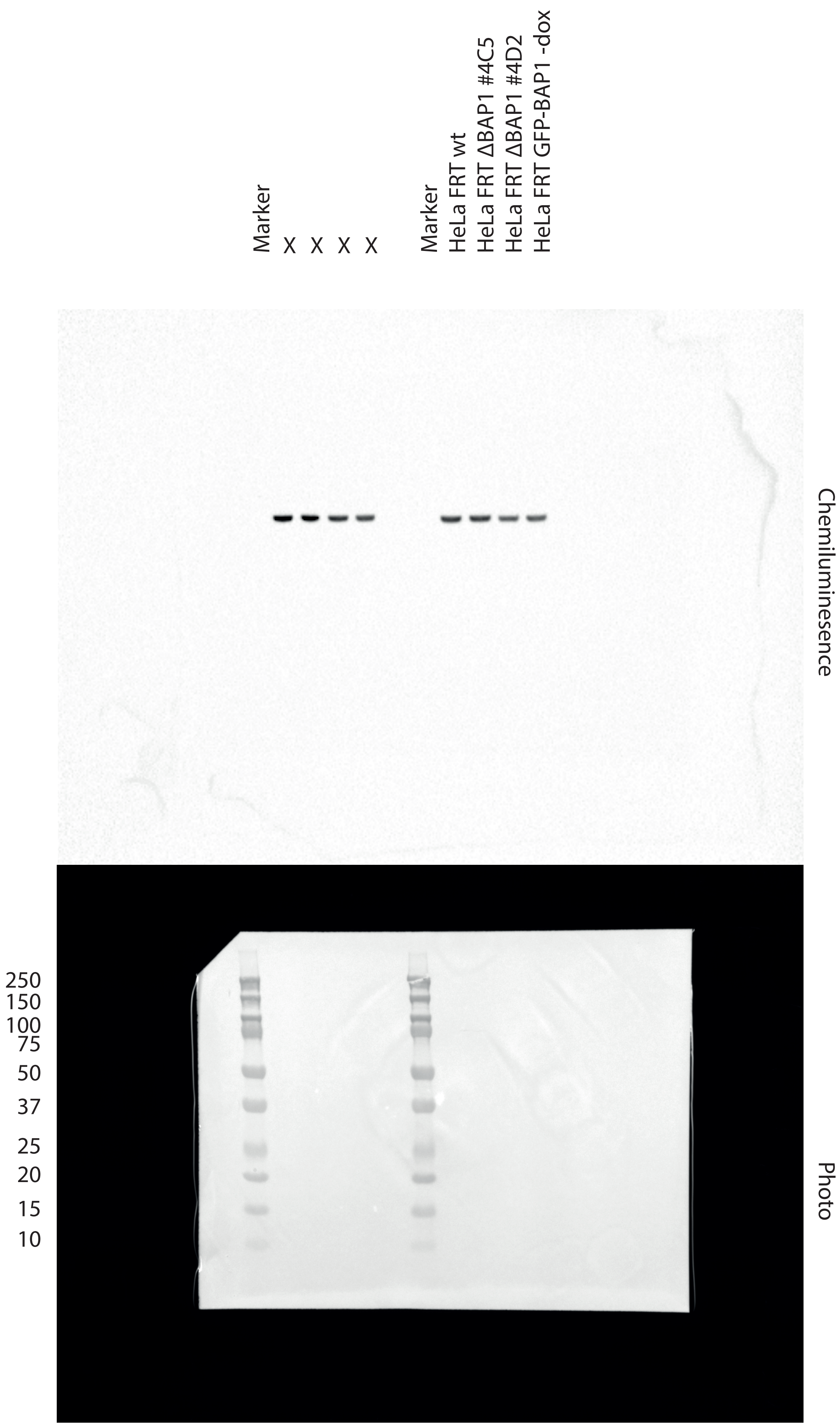
Marker
X X X X
Marker
HeLa FRT wt
HeLa FRT ΔBAP1 #4C5
HeLa FRT ΔBAP1 #4D2
HeLa FRT GFP-BAP1 -dox
Chemiluminesence
250
150
100
75
50
37
25
20
15
10
Photo
Tubulin

Fig 2A

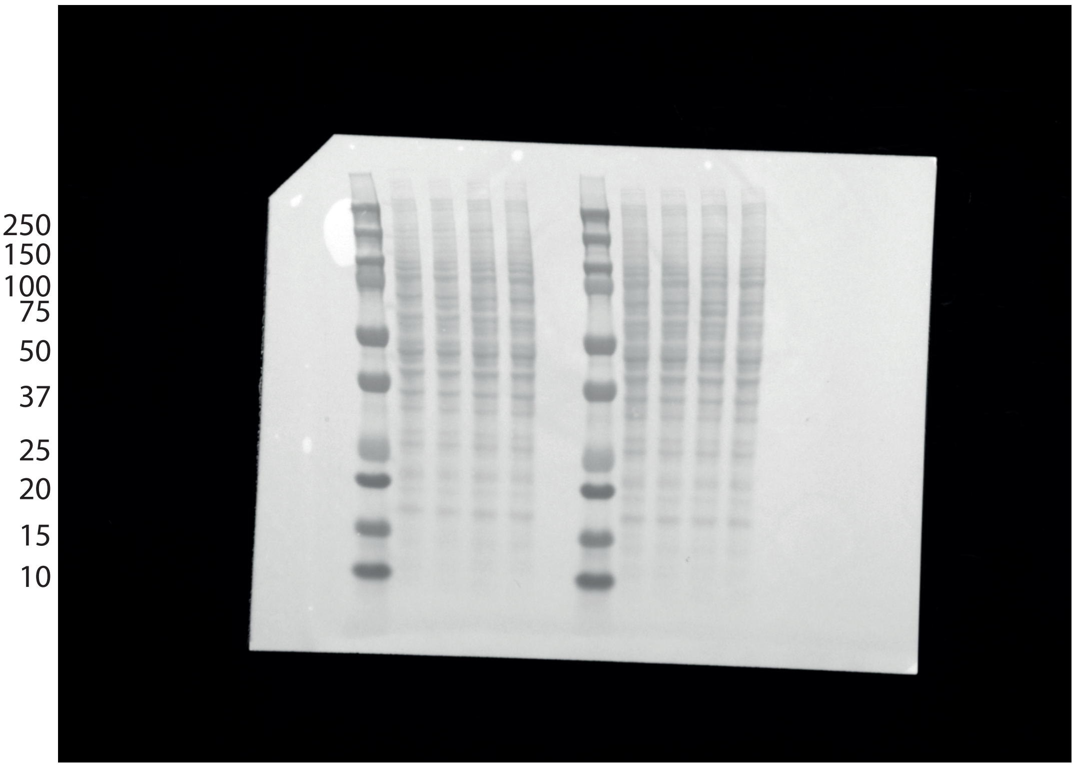

Ponceau

Fig 2B

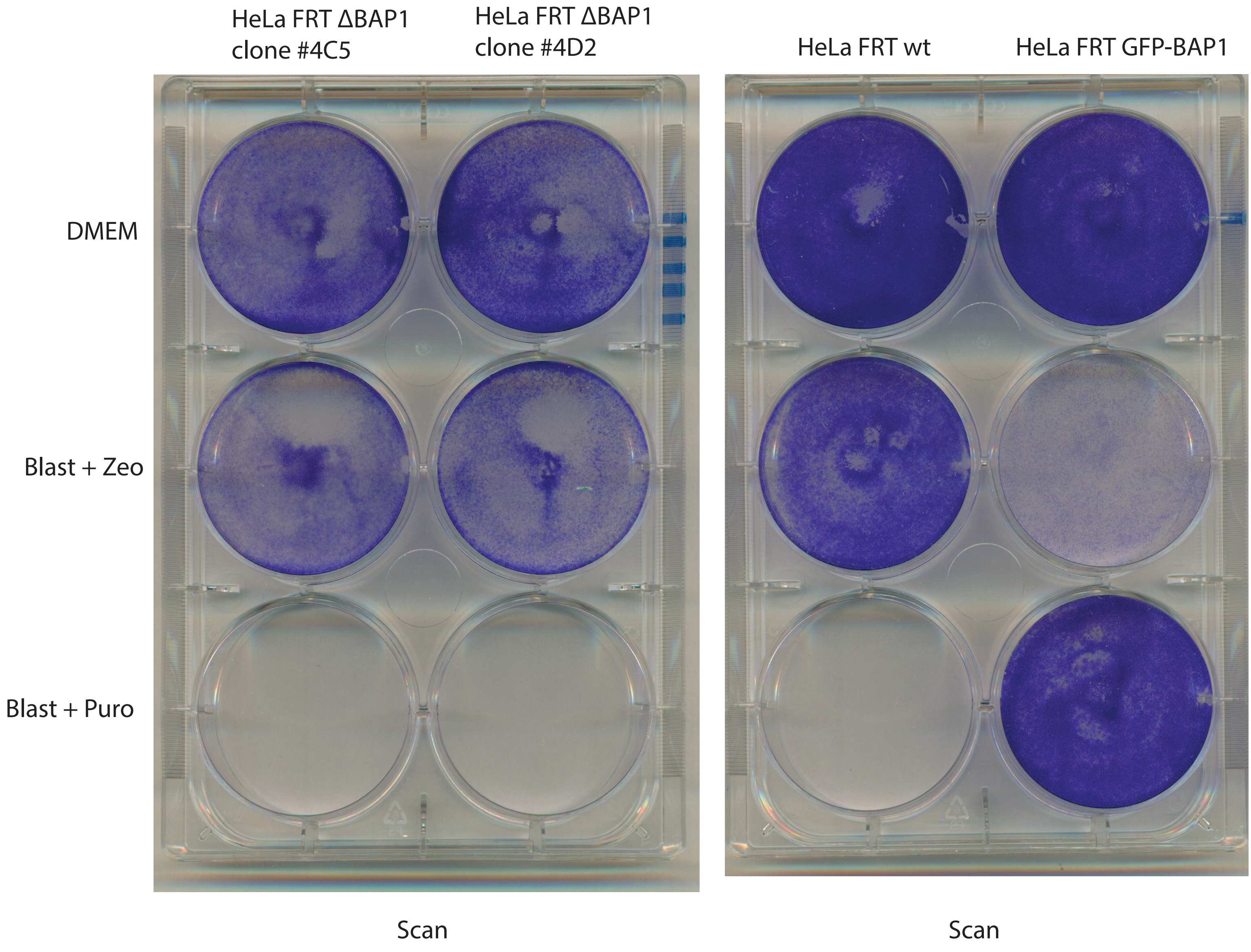
HeLa FRT ΔBAP1
clone #4C5
HeLa FRT ΔBAP1
clone #4D2
HeLa FRT wt
HeLa FRT GFP-BAP1
DMEM
Blast + Zeo
Blast + Puro
Scan
Scan

Fig 3B

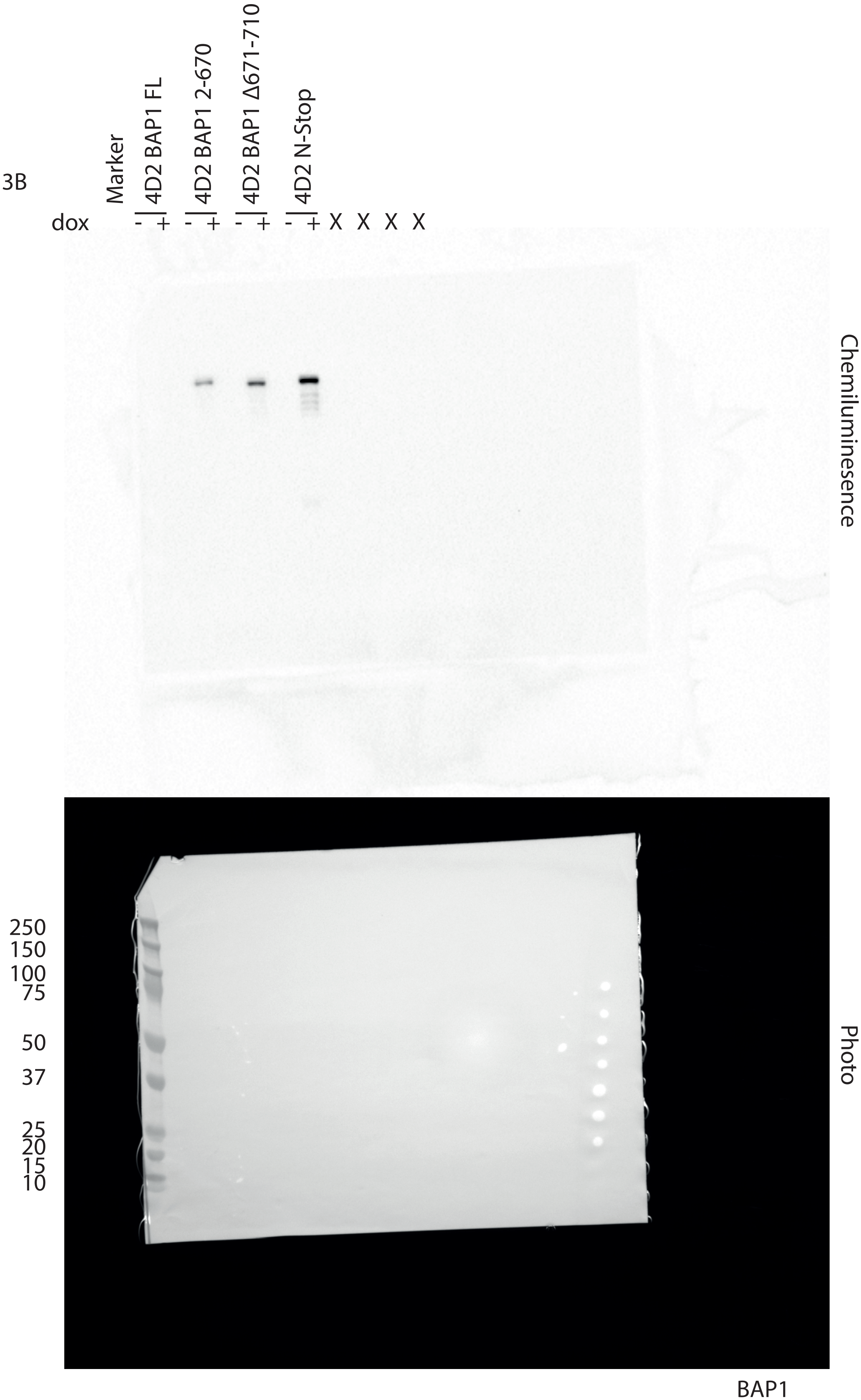

Marker
4D2 BAP1 FL
4D2 BAP1 2-670
4D2 BAP1 Δ671-710
4D2 N-Stop
dox
- + - + - + - + X X X X
Chemiluminesence
250
150
100
75
50
37
25
20
15
10
Photo

BAP1

Fig 3B

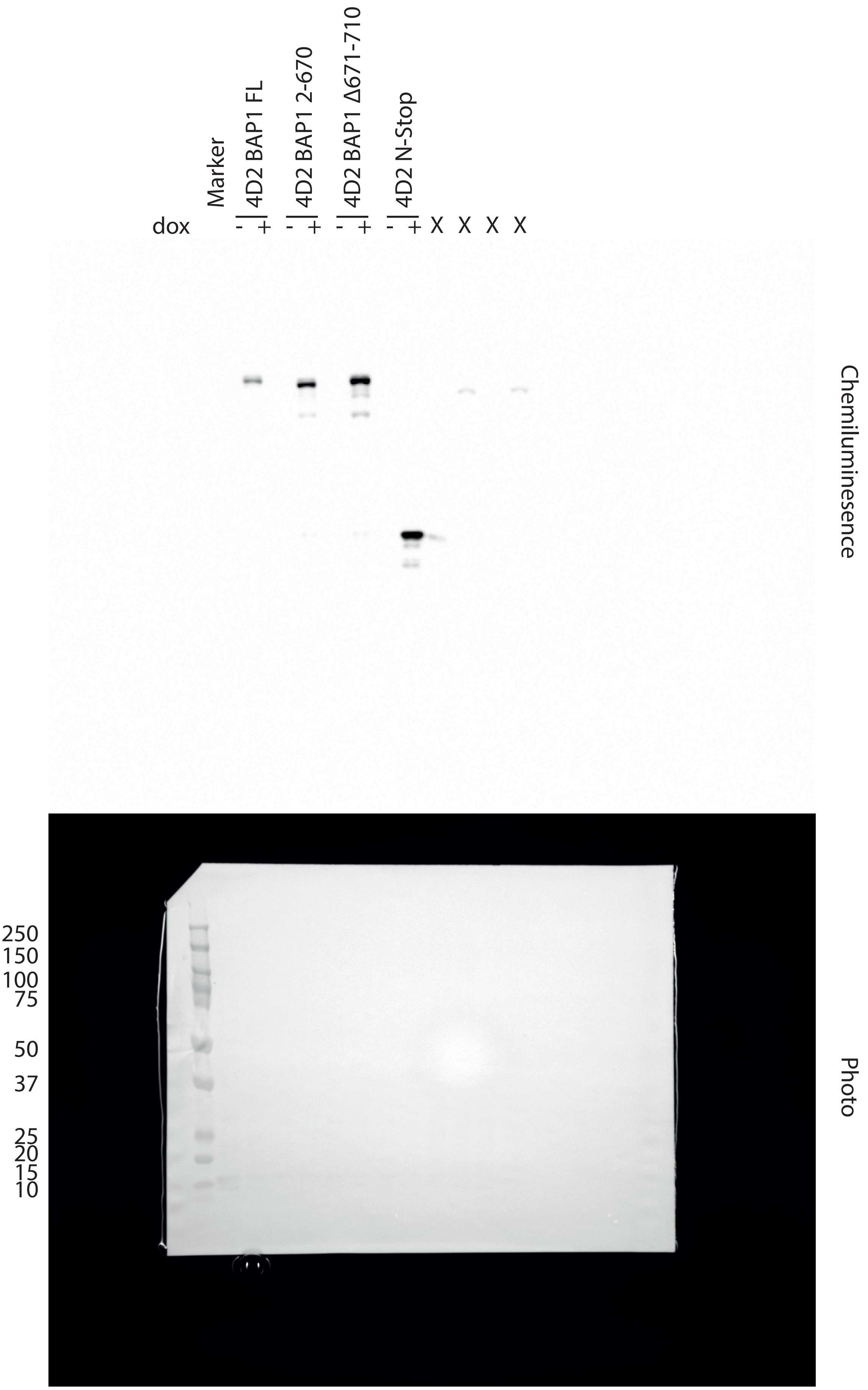
Marker
4D2 BAP1 FL
4D2 BAP1 2-670
4D2 BAP1 Δ671-710
4D2 N-Stop
dox
- + - + - + - + X X X X
Chemiluminesence
250
150
100
75
50
37
25
20
15
10
Photo

GFP

Fig 3B

Marker
4D2 BAP1 FL
4D2 BAP1 2-670
4D2 BAP1 Δ671-710
4D2 N-Stop

dox - + - + - + - + X X X X

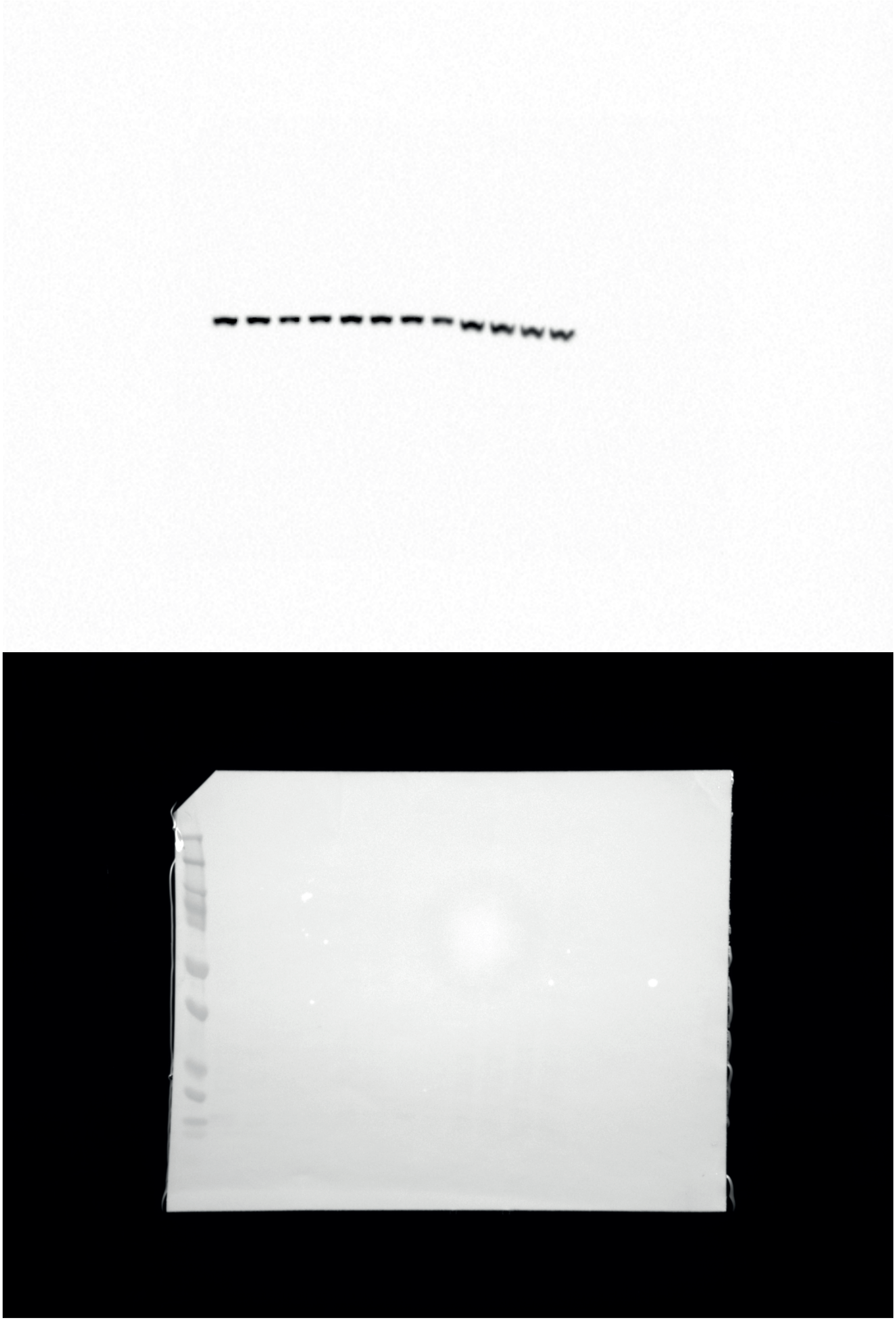

Chemiluminesence

250
150
100
75
50
37
25
20
15
10

Photo

Tubulin

Fig 3B

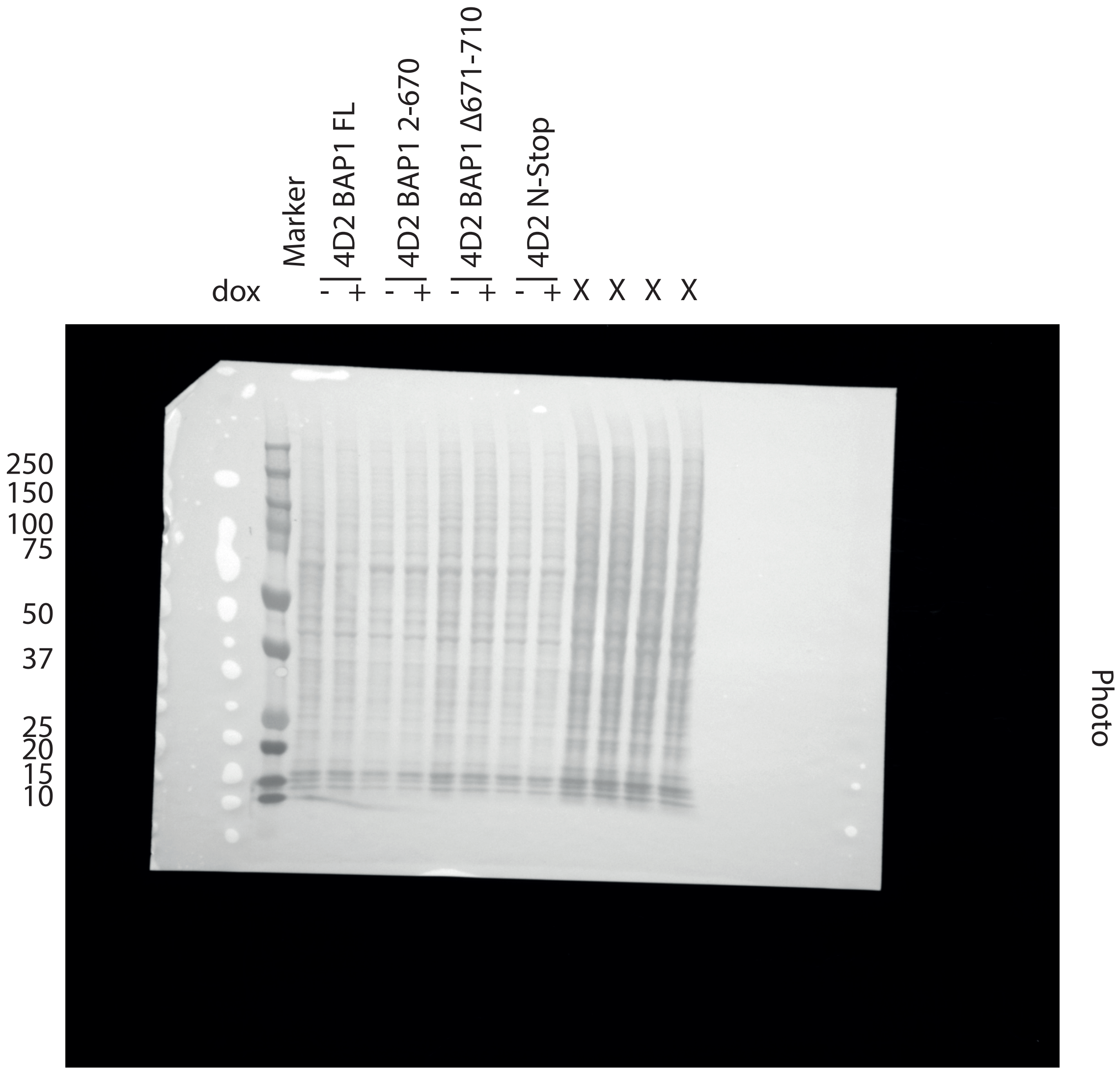

Ponceau

Fig 5B

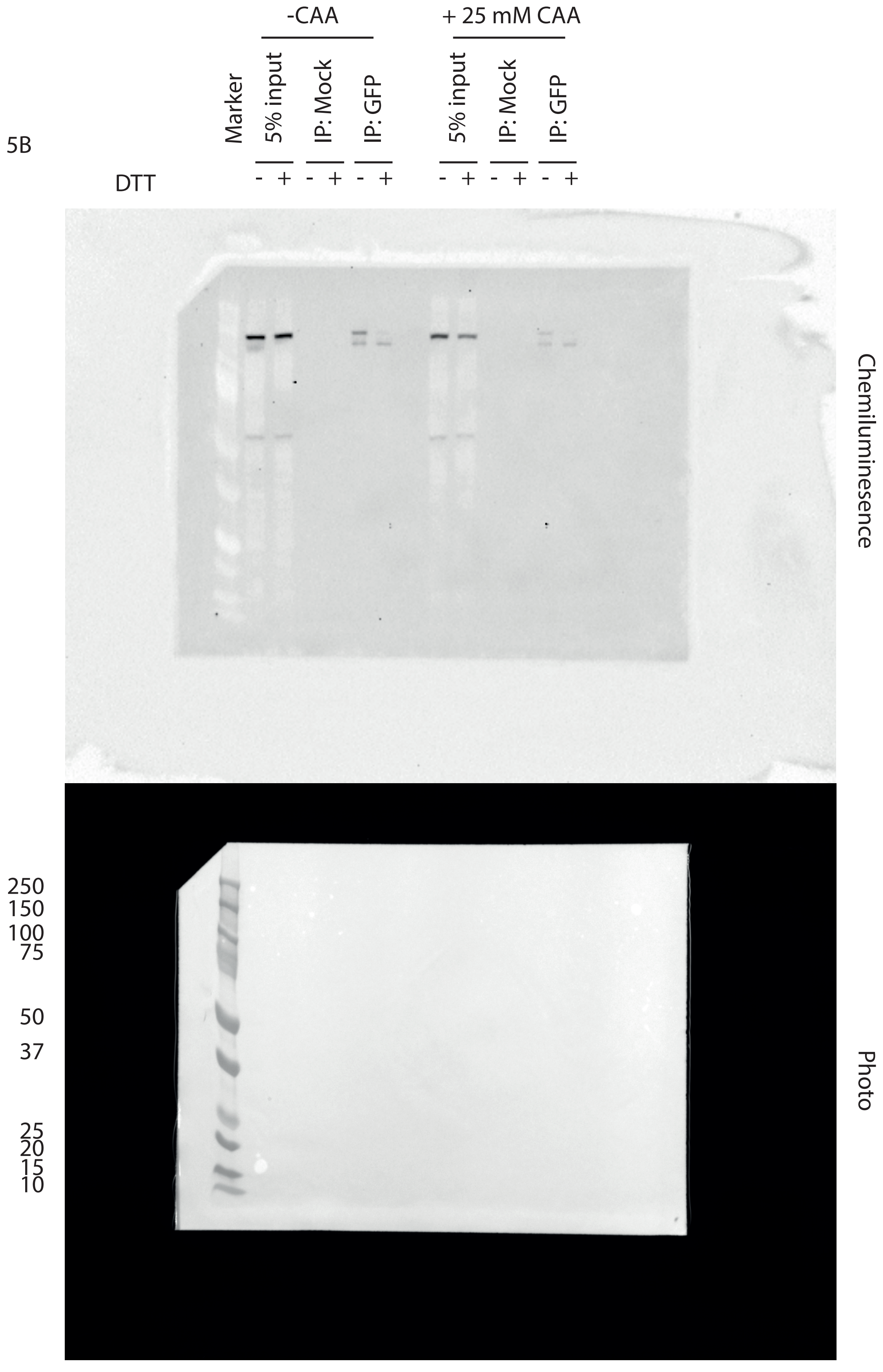
-CAA
+ 25 mM CAA
Marker
5% input
IP: Mock
IP: GFP
5% input
IP: Mock
IP: GFP
DTT
- + - + - + - + - + - +
Chemiluminesence
250
150
100
75
50
37
25
20
15
10
Photo
COPA

Fig 5B

-CAA | + 25 mM CAA

Marker | 5% input | IP: Mock | IP: GFP | 5% input | IP: Mock | IP: GFP

DTT - + - + - + - + - + - +

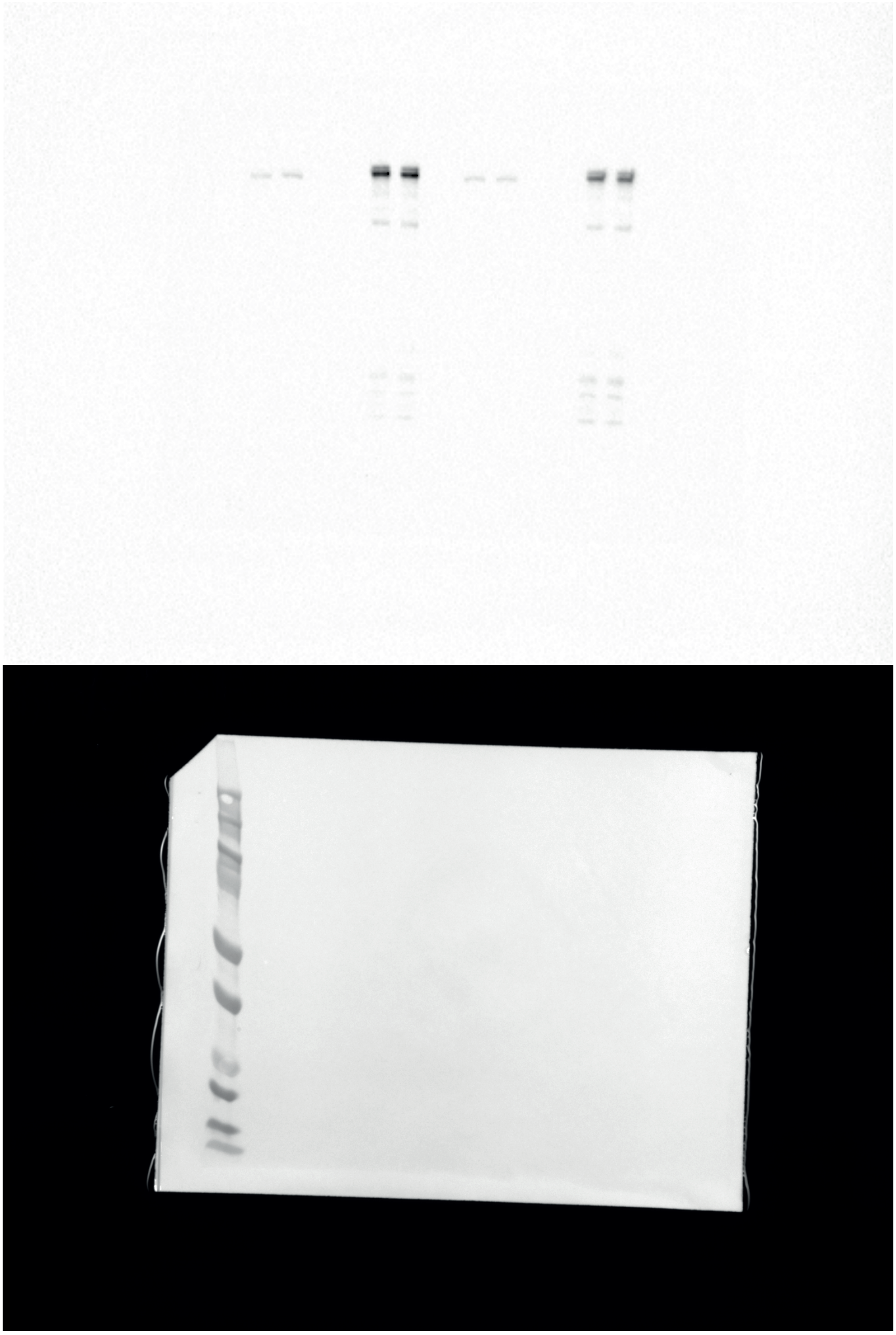

Chemiluminesence

250
150
100
75
50
37
25
20
15
10

Photo

GFP

Fig 5B

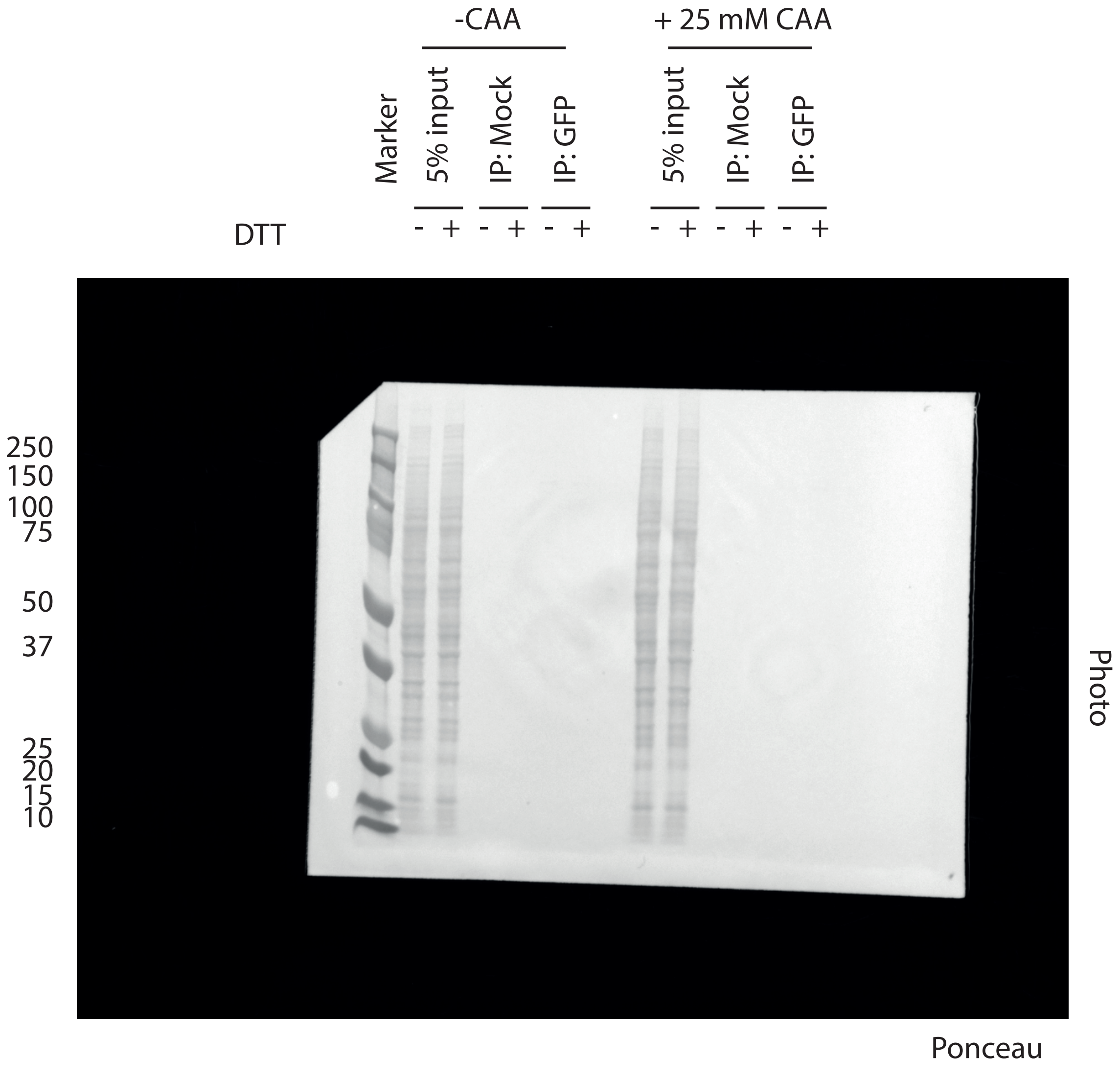
-CAA
+ 25 mM CAA
Marker
5% input
IP: Mock
IP: GFP
5% input
IP: Mock
IP: GFP
DTT
- + - + - +
- + - + - +
250
150
100
75
50
37
25
20
15
10
Photo
Ponceau

Fig 5C

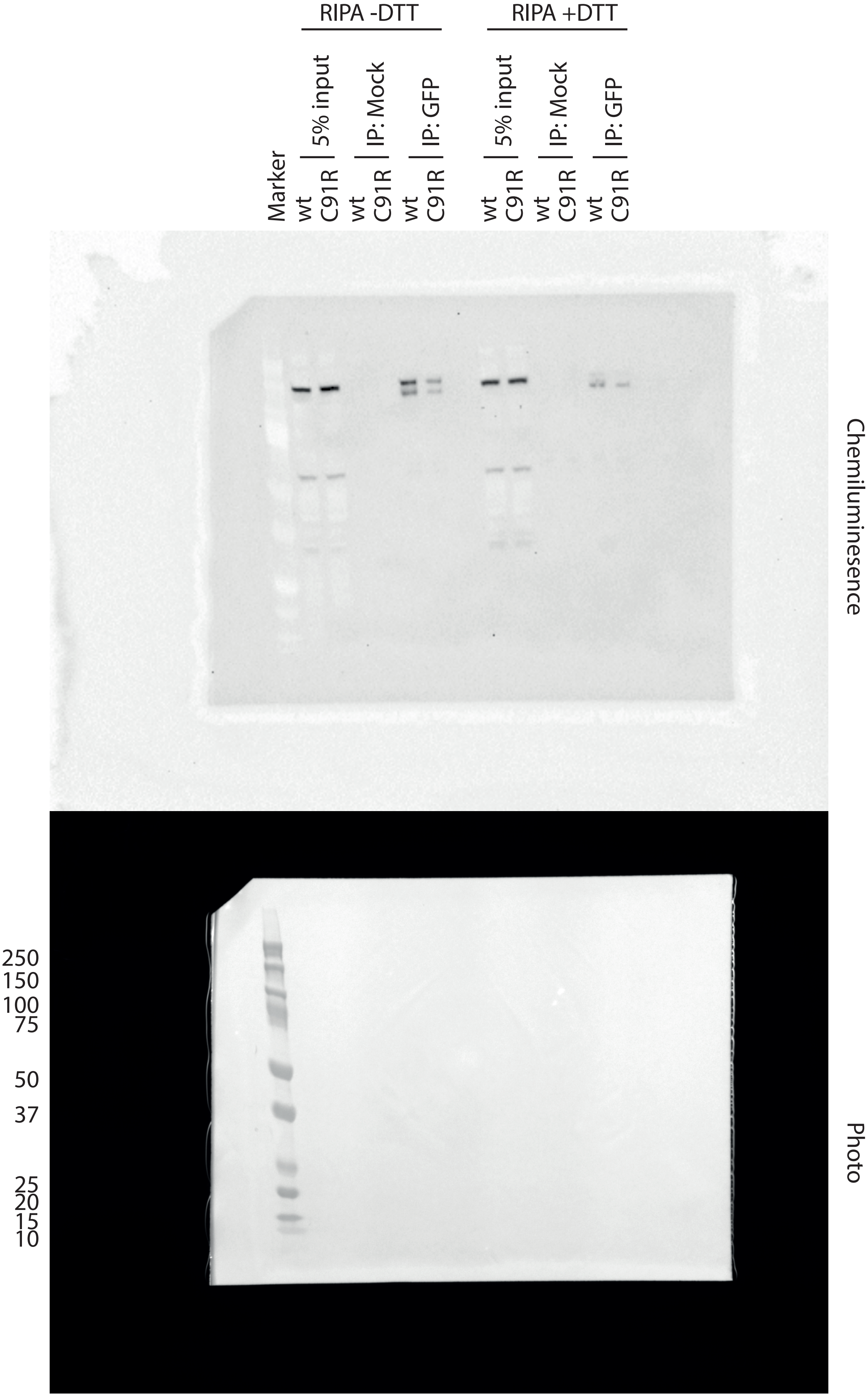

RIPA -DTT
RIPA +DTT
5% input
IP: Mock
IP: GFP
5% input
IP: Mock
IP: GFP
Marker
wt
C91R
wt
C91R
wt
C91R
wt
C91R
wt
C91R
wt
C91R
Chemiluminesence
Photo
250
150
100
75
50
37
25
20
15
10

COPA

Fig 5C

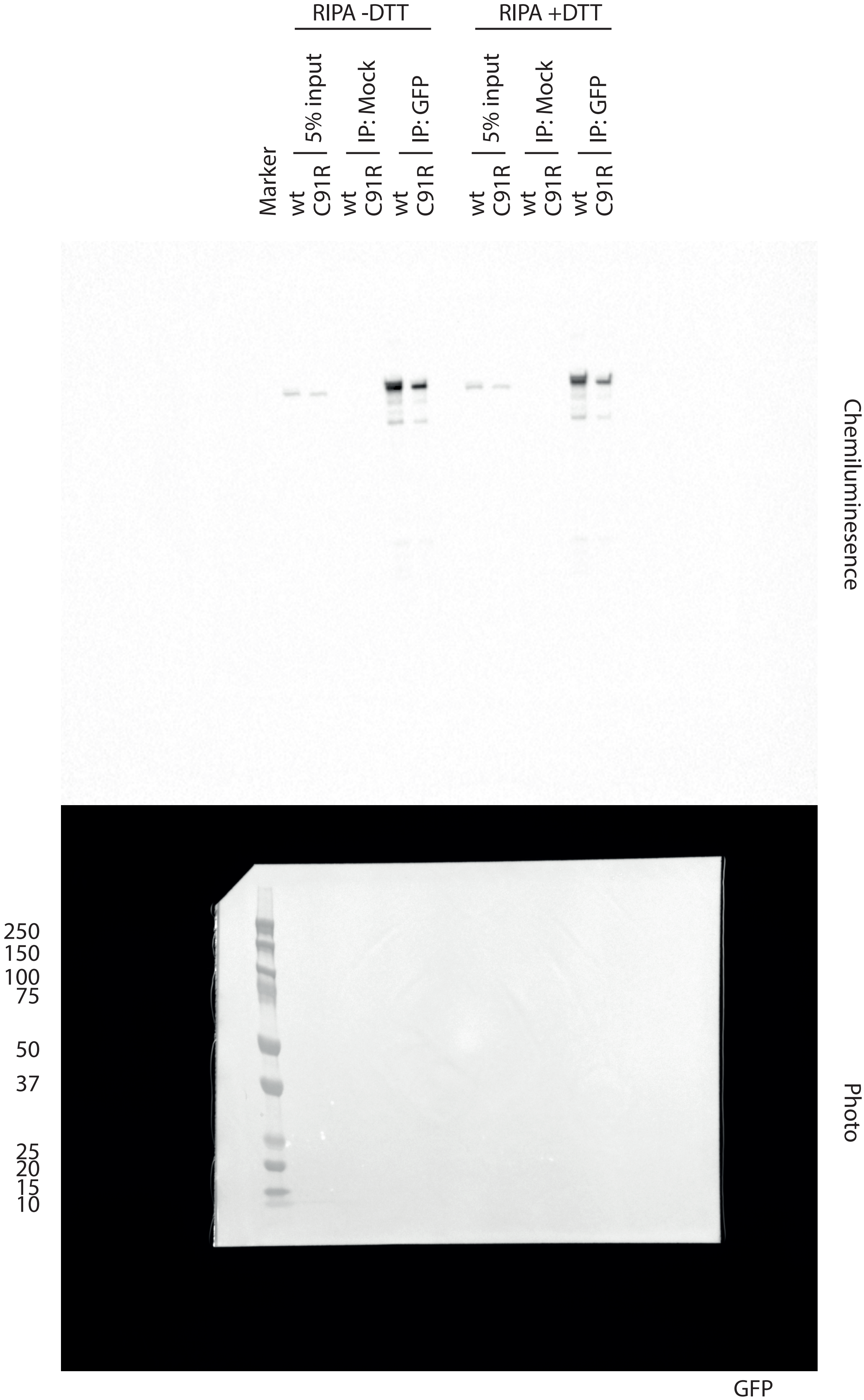

RIPA -DTT
RIPA +DTT
5% input
IP: Mock
IP: GFP
5% input
IP: Mock
IP: GFP
Marker
wt
C91R
wt
C91R
wt
C91R
wt
C91R
wt
C91R
wt
C91R
Chemiluminesence
250
150
100
75
50
37
25
20
15
10
Photo
GFP

Fig 5C

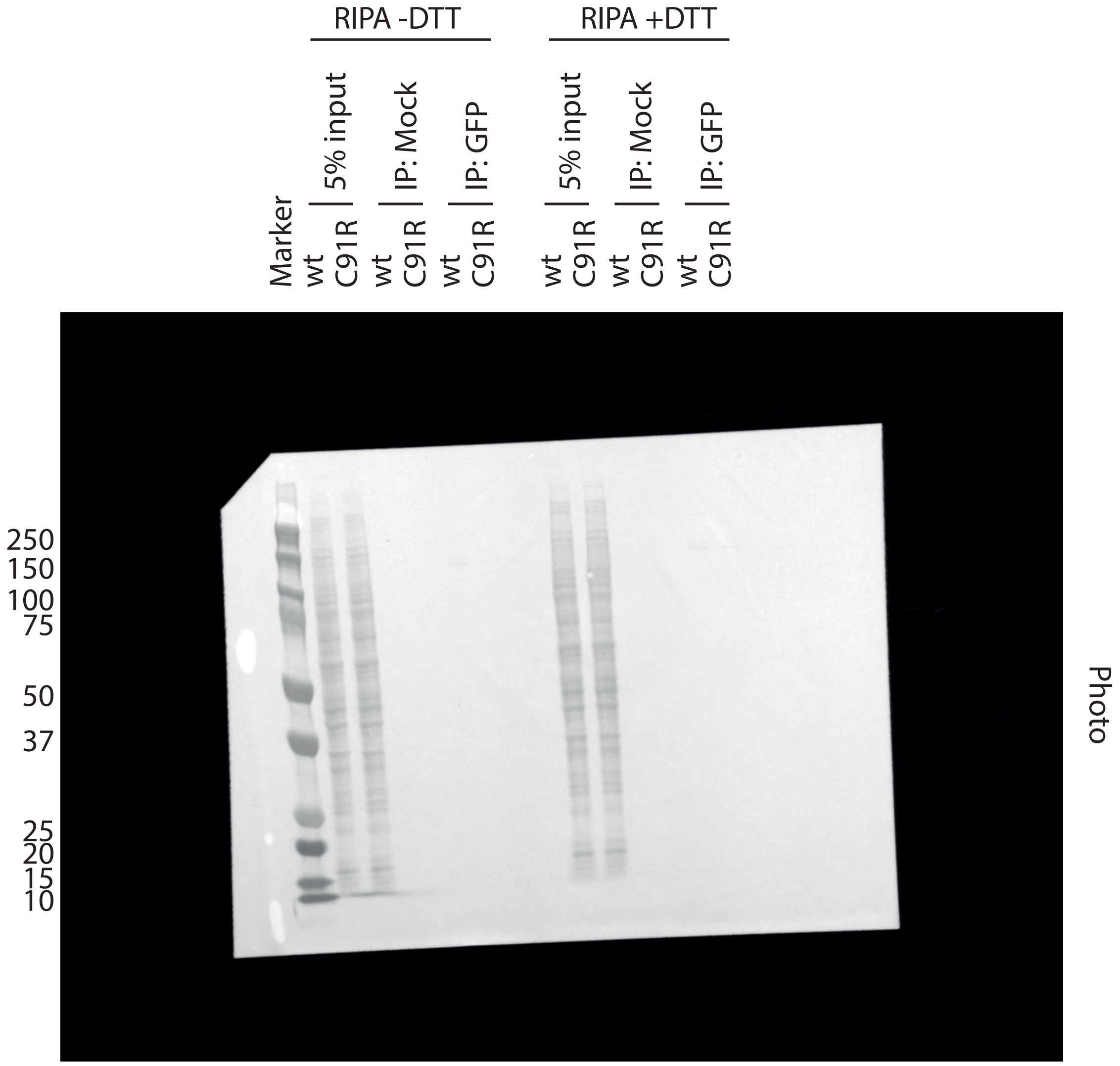

Ponceau

Fig 5D

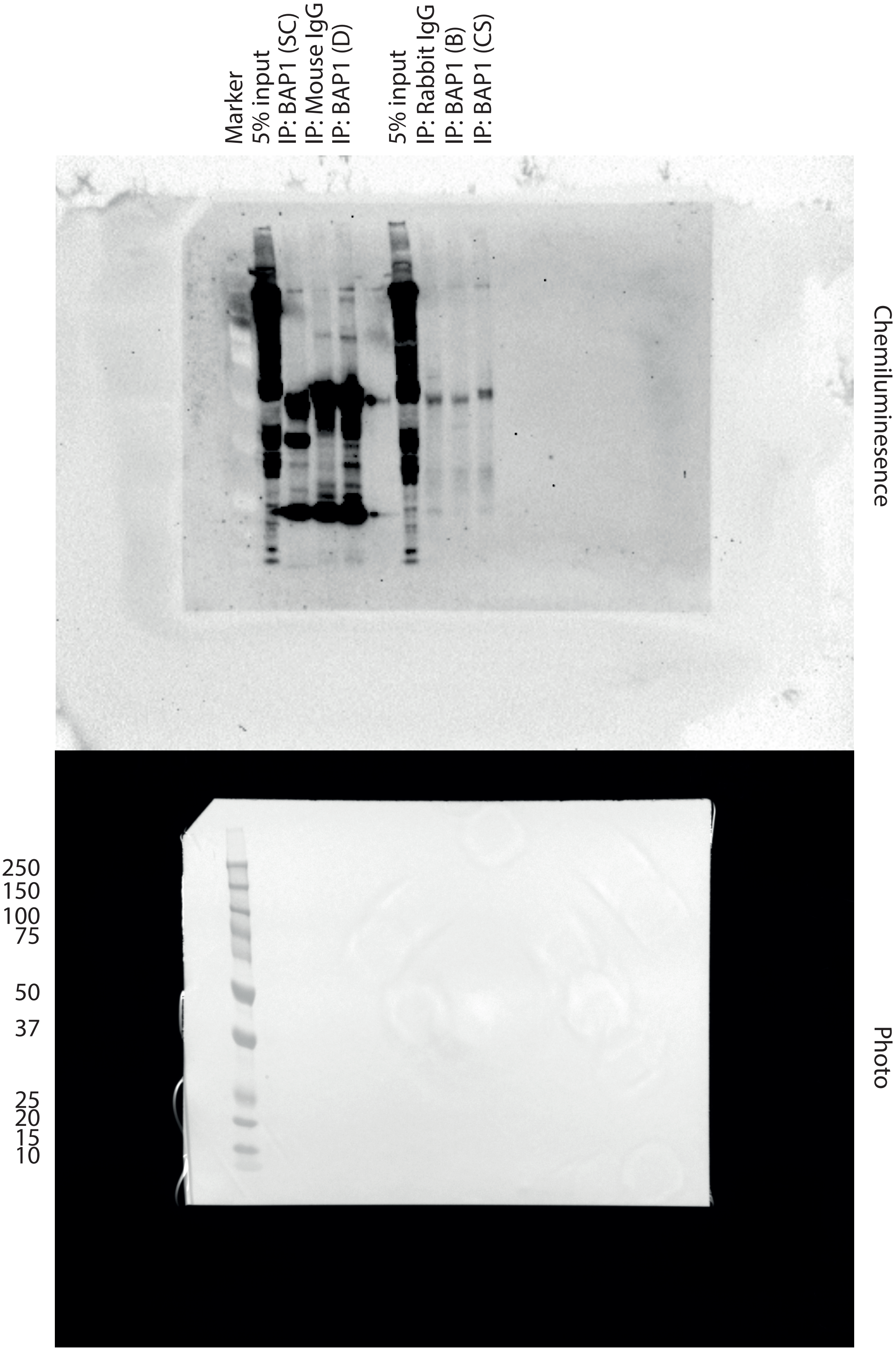
Marker
5% input
IP: BAP1 (SC)
IP: Mouse IgG
IP: BAP1 (D)
5% input
IP: Rabbit IgG
IP: BAP1 (B)
IP: BAP1 (CS)
Chemiluminesence
250
150
100
75
50
37
25
20
15
10
Photo

COPA

Fig 5D

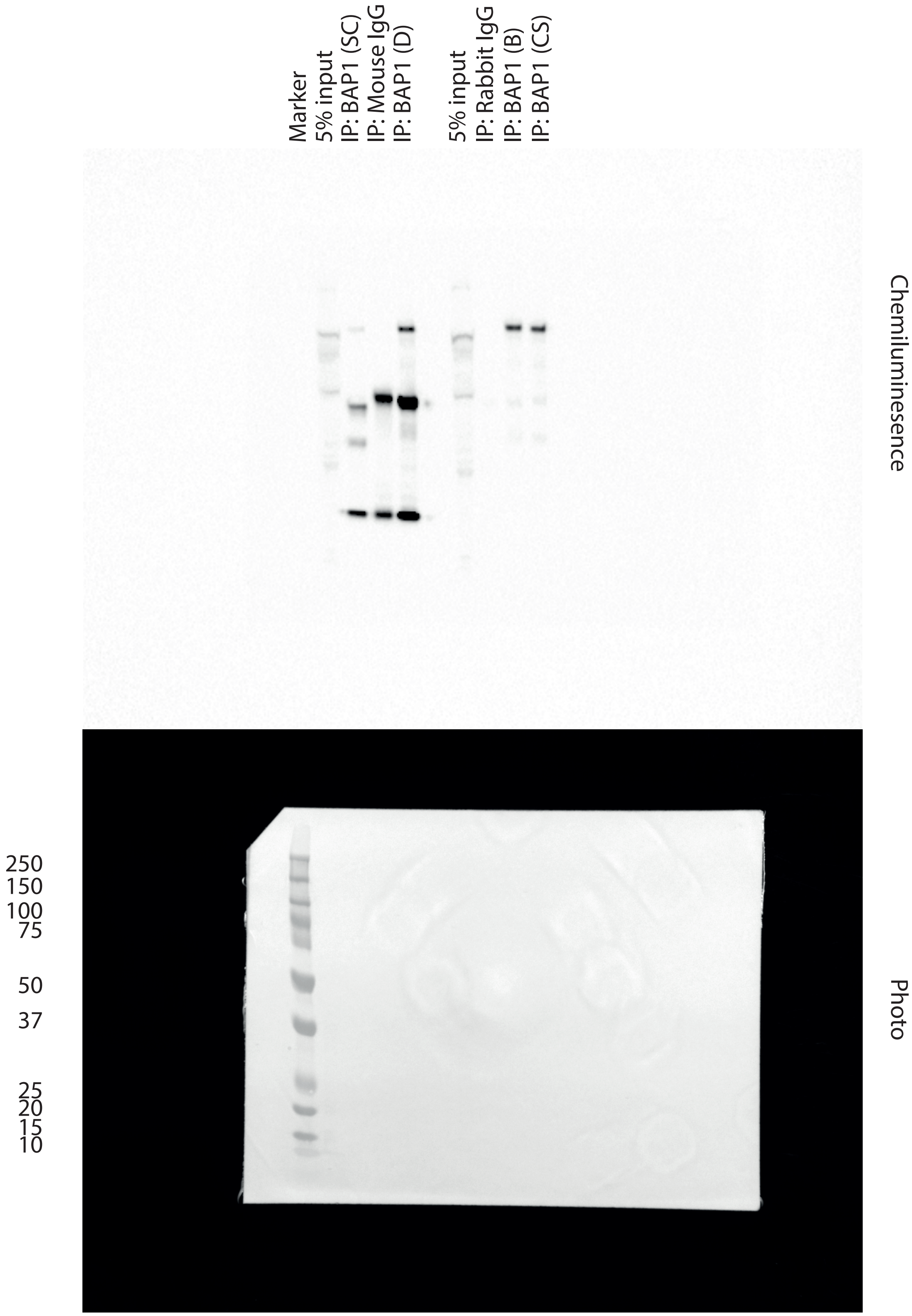
Marker
5% input
IP: BAP1 (SC)
IP: Mouse IgG
IP: BAP1 (D)
5% input
IP: Rabbit IgG
IP: BAP1 (B)
IP: BAP1 (CS)
Chemiluminesence
250
150
100
75
50
37
25
20
15
10
Photo

BAP1

Fig 5D

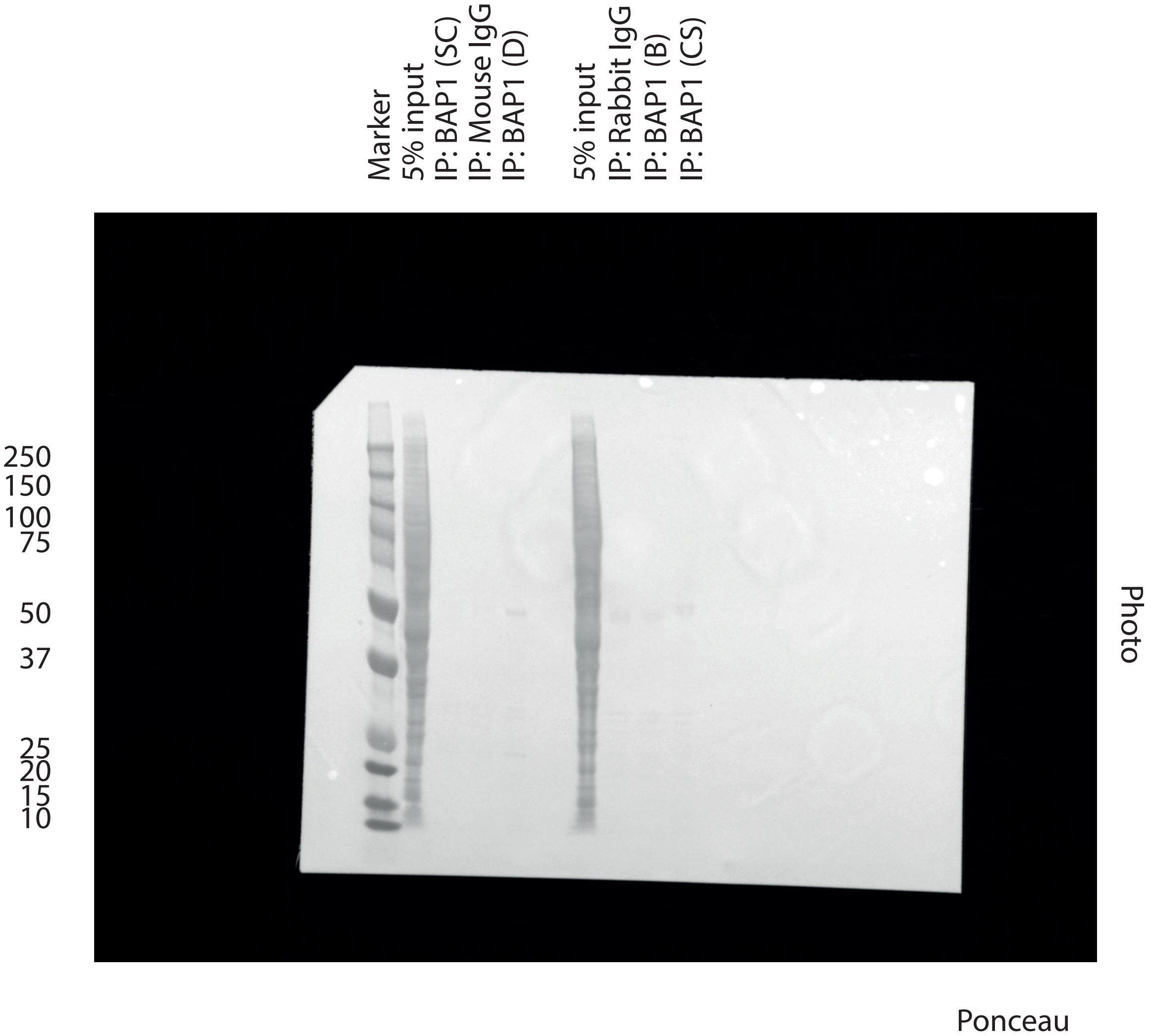

Ponceau

Fig 5E

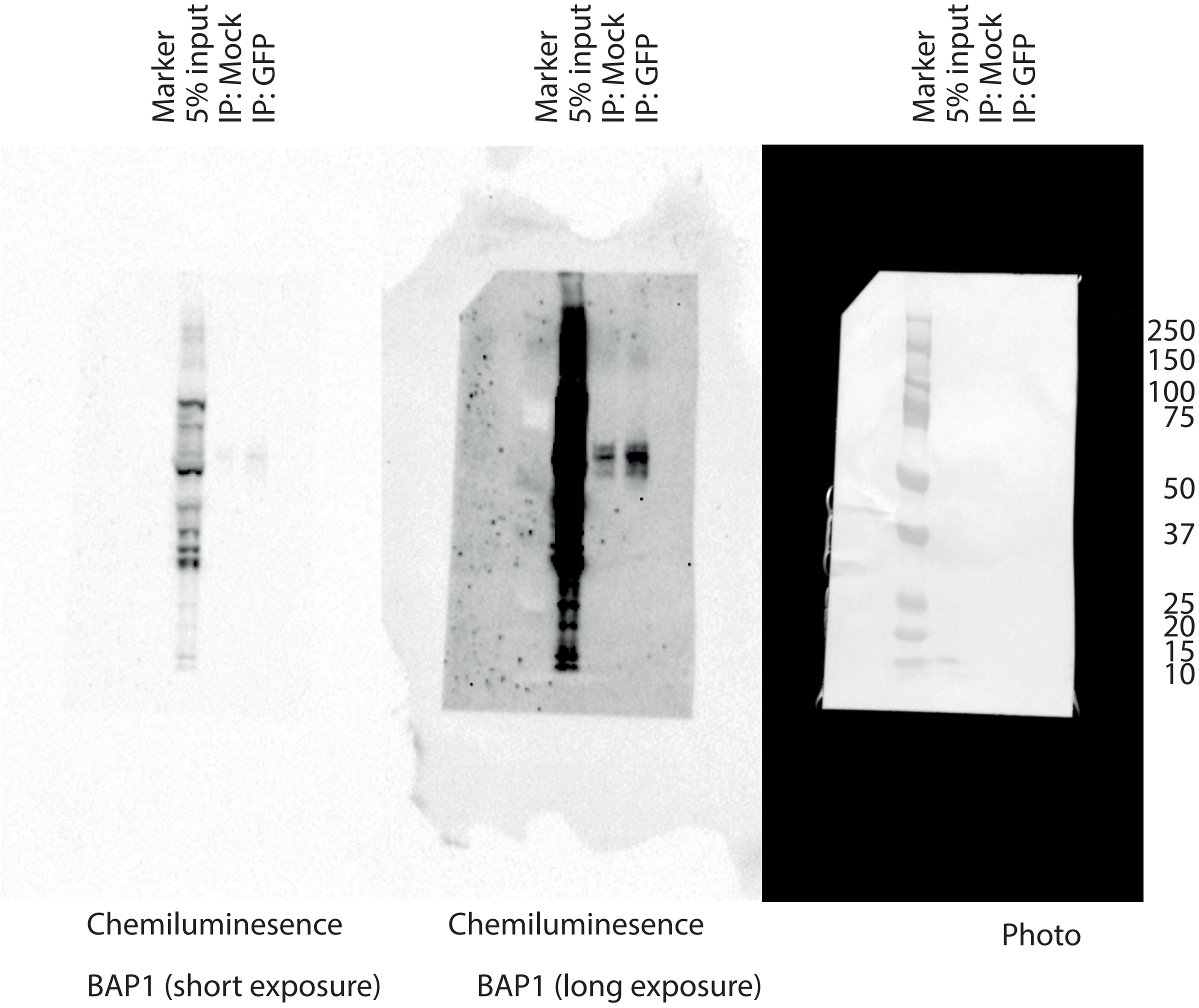
Marker
5% input
IP: Mock
IP: GFP
Marker
5% input
IP: Mock
IP: GFP
Marker
5% input
IP: Mock
IP: GFP
250
150
100
75
50
37
25
20
15
10
Chemiluminesence
Chemiluminesence
Photo
BAP1 (short exposure)
BAP1 (long exposure)

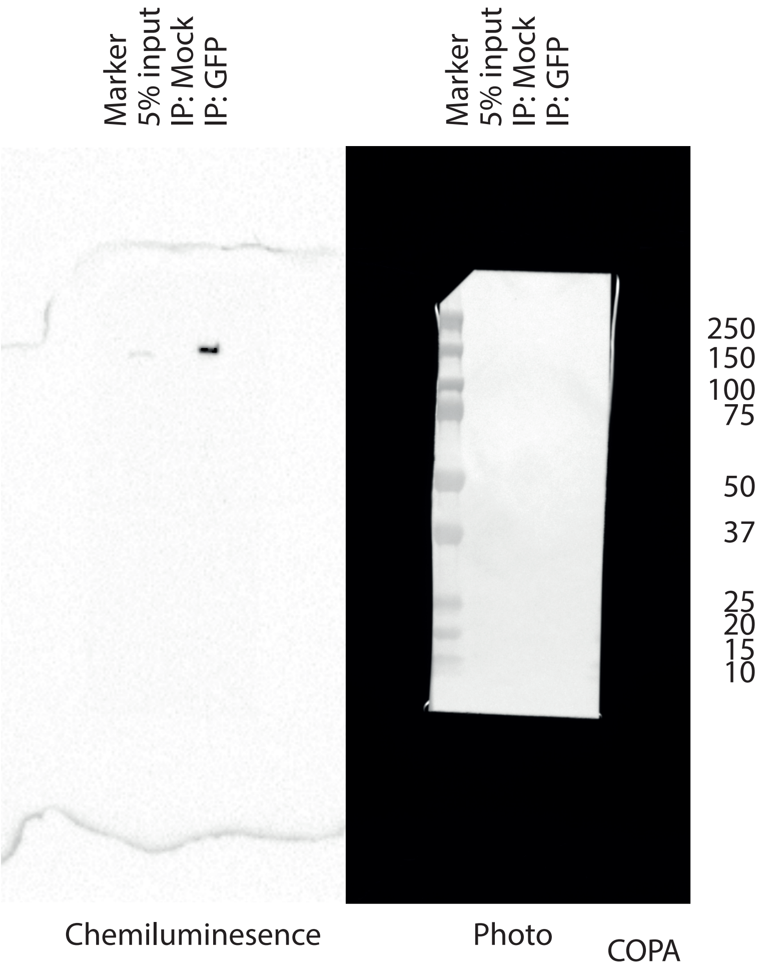
Marker
5% input
IP: Mock
IP: GFP
Marker
5% input
IP: Mock
IP: GFP
250
150
100
75
50
37
25
20
15
10
Chemiluminesence
Photo
COPA

Fig 5E

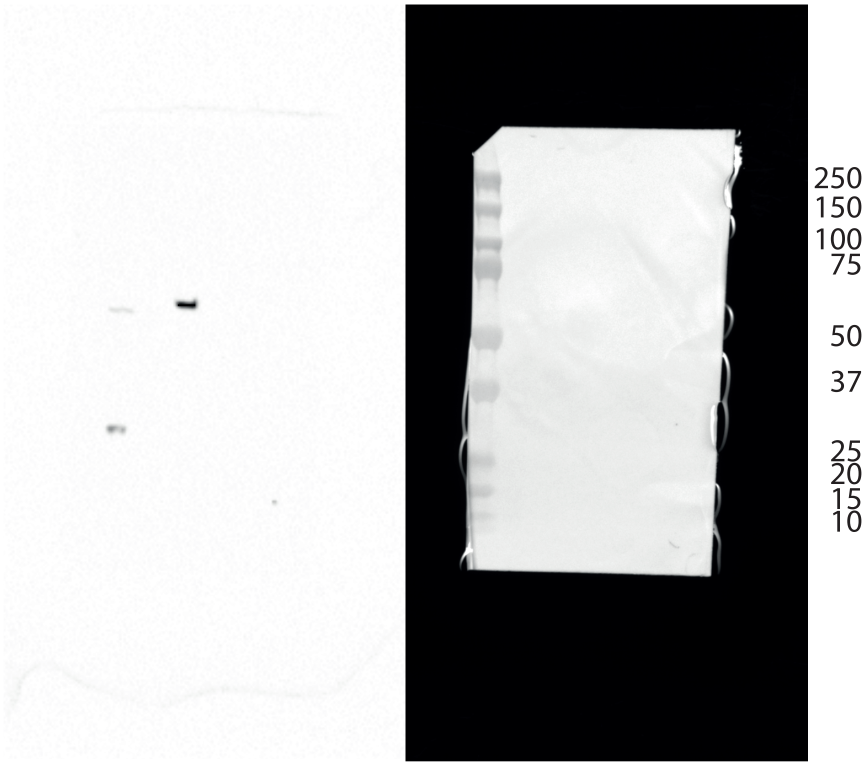

Marker
5% input
IP: Mock
IP: GFP
Marker
5% input
IP: Mock
IP: GFP
250
150
100
75
50
37
25
20
15
10
Chemiluminesence
Photo
COPE

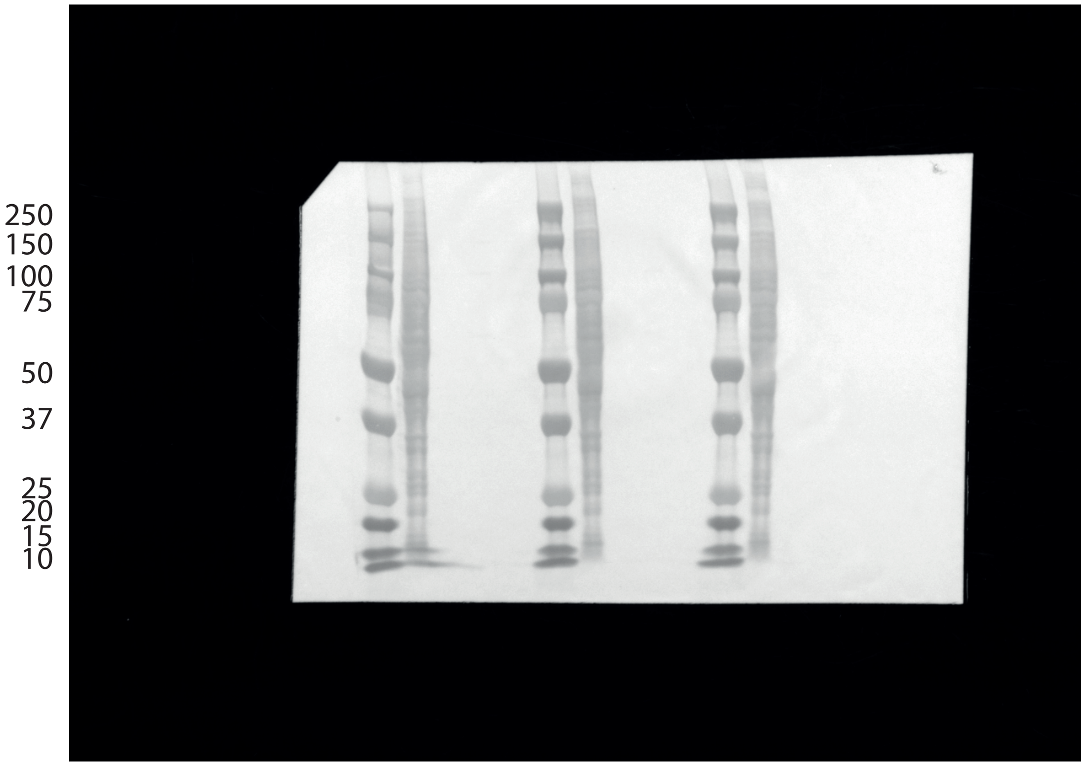

Marker
5% input
IP: Mock
IP: GFP
X X X X
X X X X
250
150
100
75
50
37
25
20
15
10
Photo
Ponceau

Fig 5F

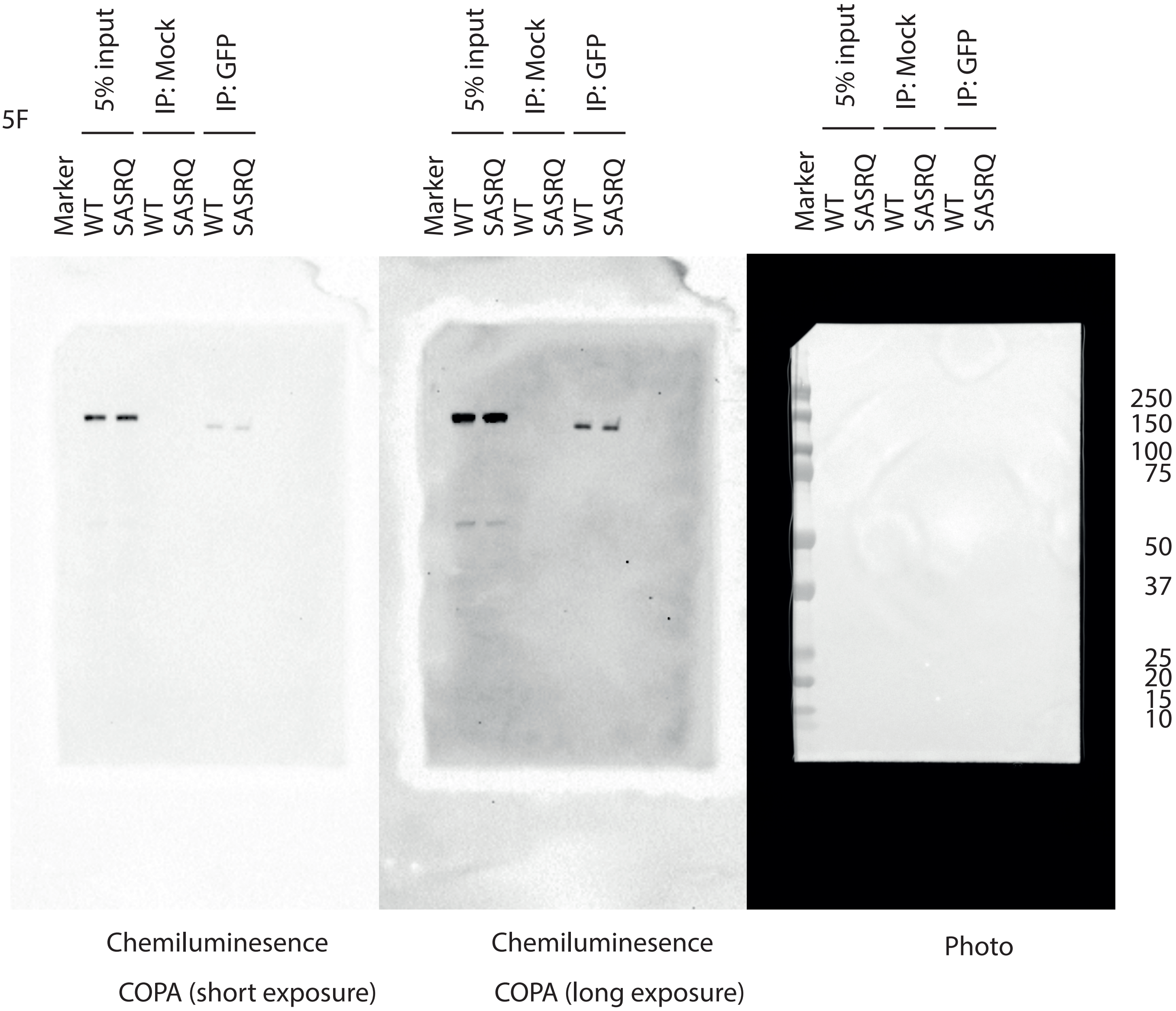
5% input
IP: Mock
IP: GFP
Marker
WT
SASRQ
WT
SASRQ
WT
SASRQ
5% input
IP: Mock
IP: GFP
Marker
WT
SASRQ
WT
SASRQ
WT
SASRQ
5% input
IP: Mock
IP: GFP
Marker
WT
SASRQ
WT
SASRQ
WT
SASRQ
250
150
100
75
50
37
25
20
15
10
Chemiluminesence
COPA (short exposure)
Chemiluminesence
COPA (long exposure)
Photo

Fig 5F

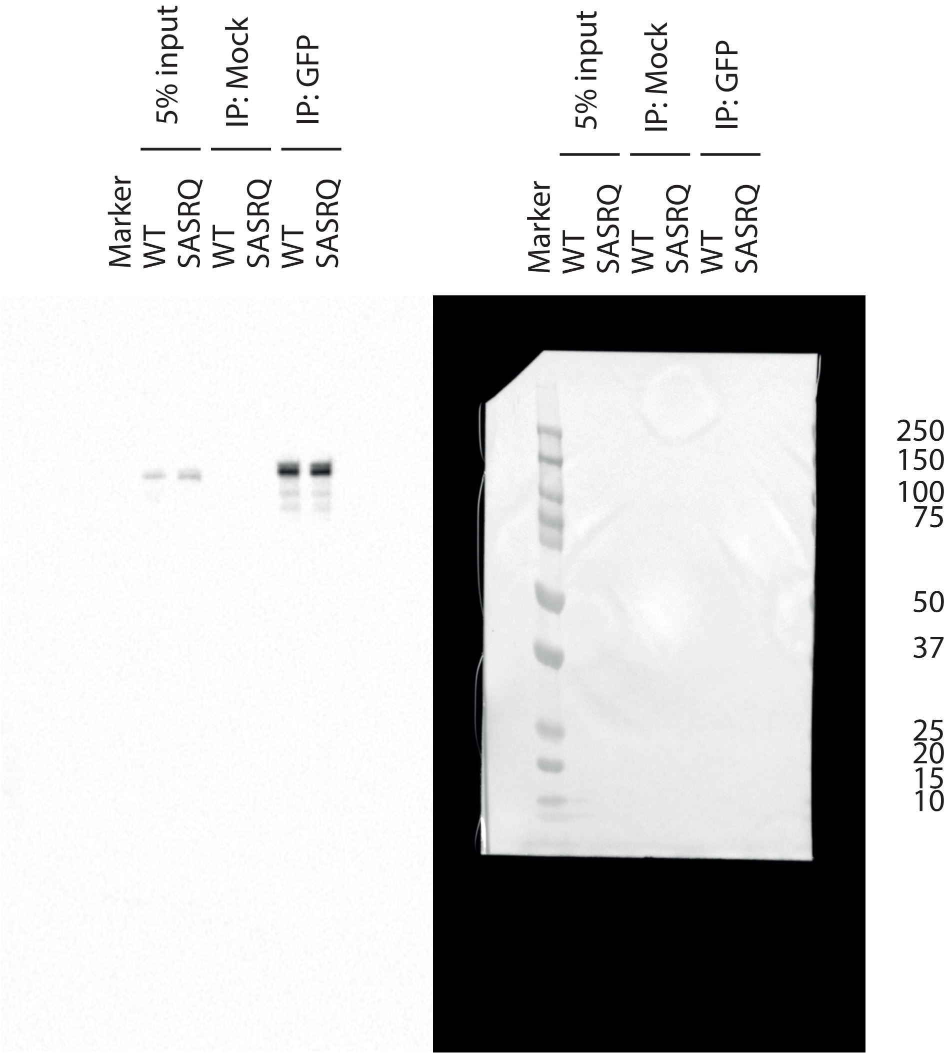

Chemiluminesence

Photo

BAP1

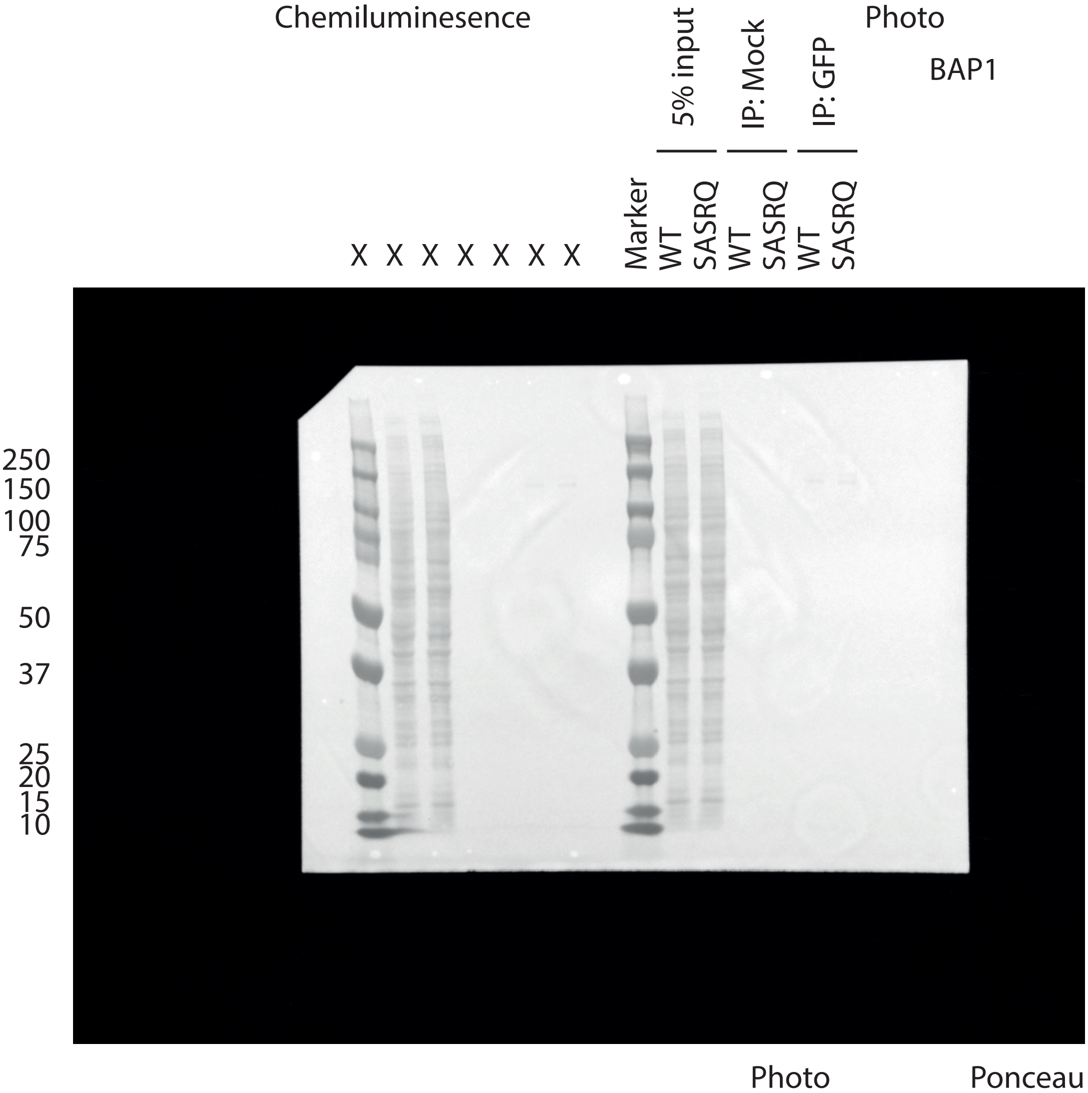

Photo

Ponceau

Fig S1A

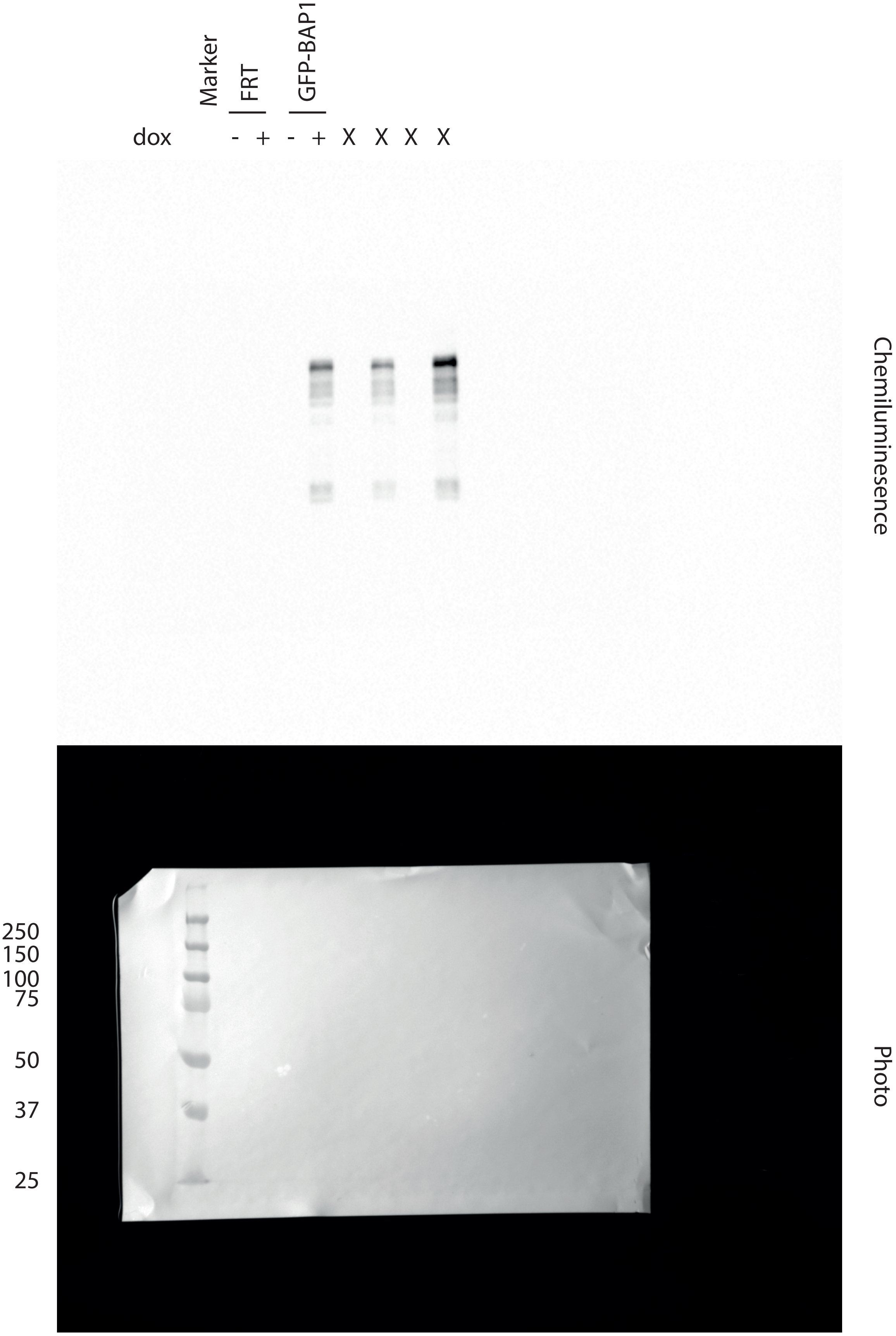

Marker
FRT
GFP-BAP1
dox
- + - + X X X X
Chemiluminesence
250
150
100
75
50
37
25
Photo

BAP1

Fig S1A

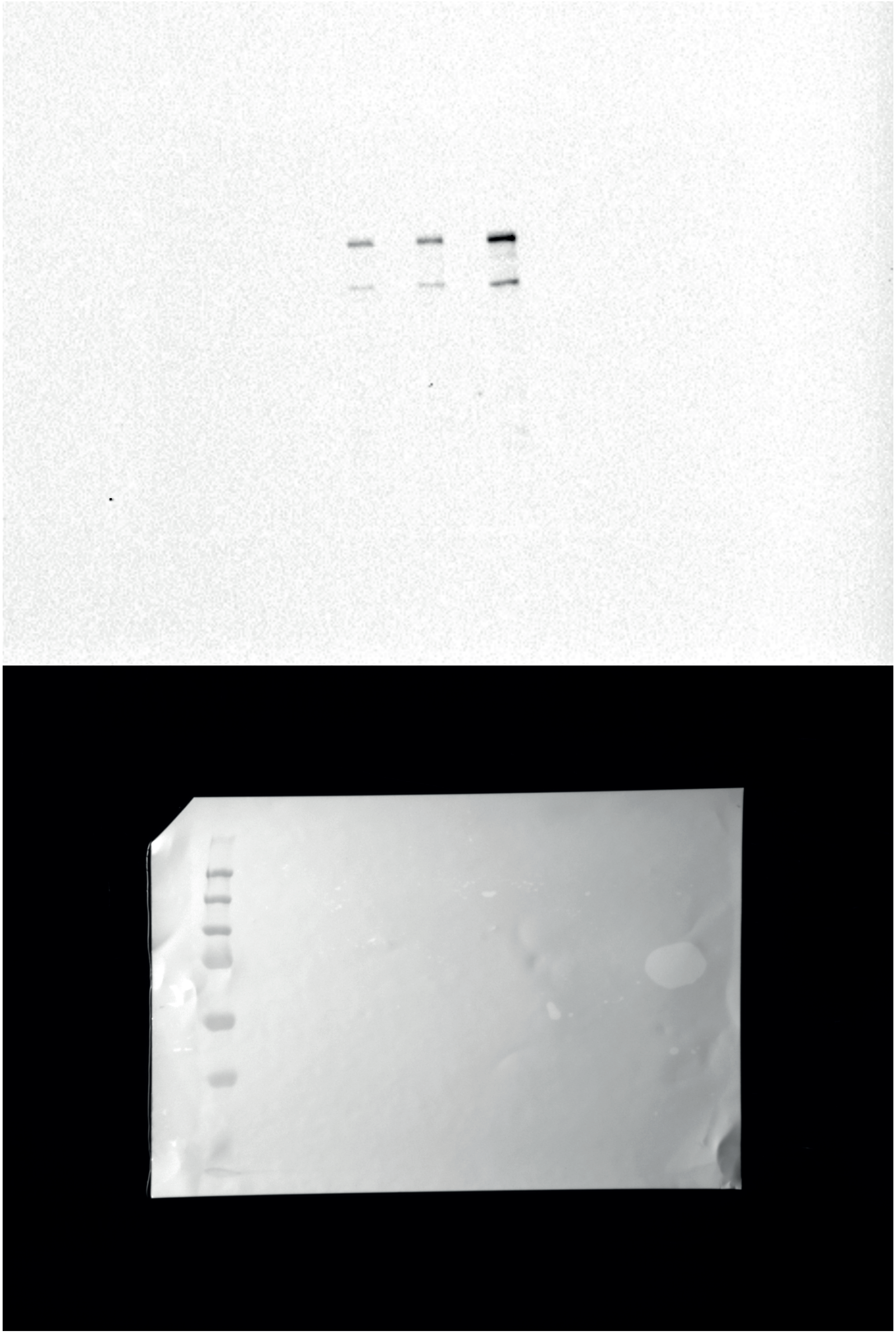
Marker
FRT
GFP-BAP1
dox
- + - + X X X X
Chemiluminesence
Photo
250
150
100
75
50
37
25
GFP

Fig S1A

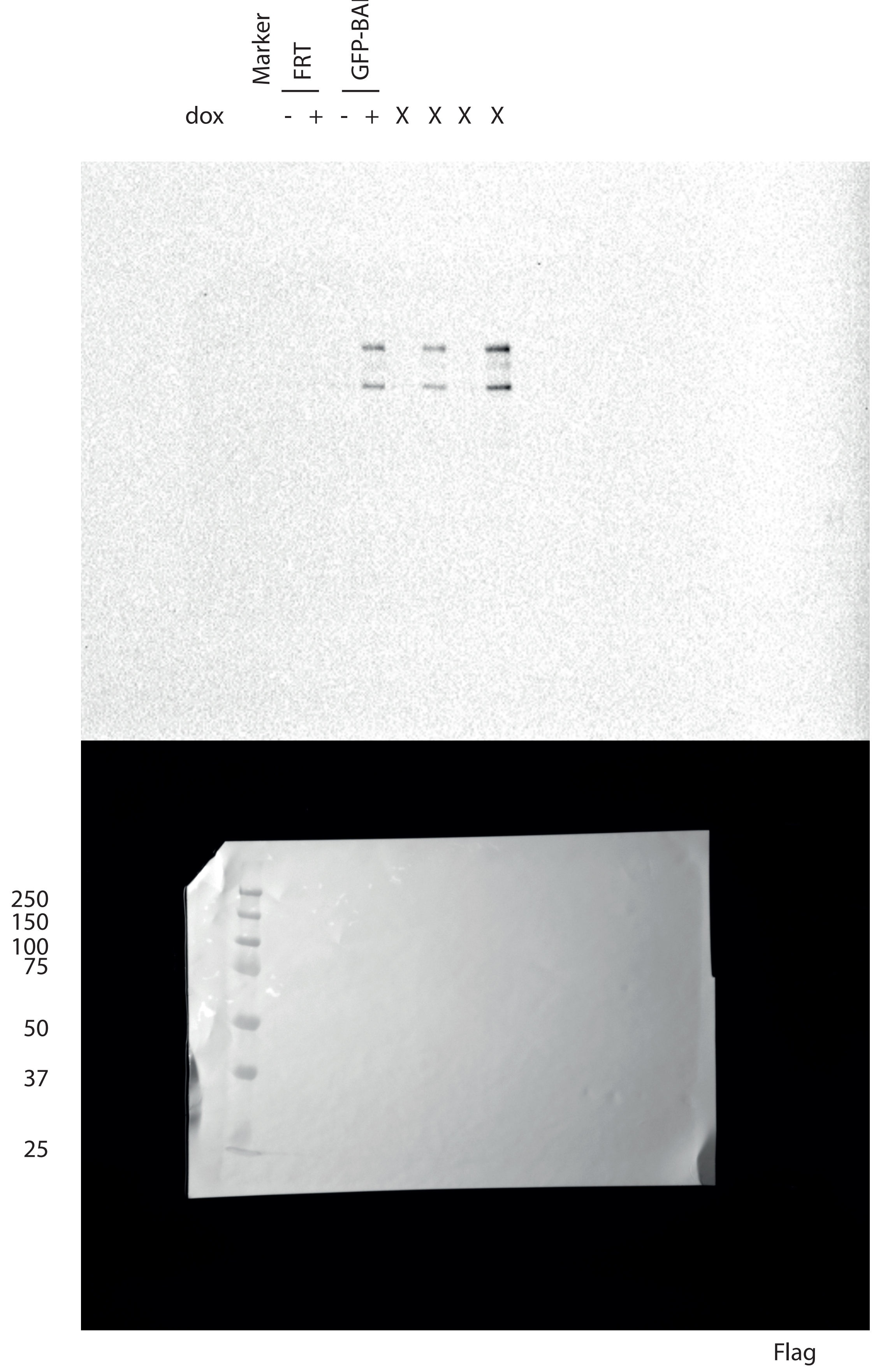
Marker
FRT
GFP-BAP1
dox
- + - + X X X X
Chemiluminesence
250
150
100
75
50
37
25
Photo
Flag

Fig S1A

Marker | FRT | GFP-BAP1

dox - + - + X X X X

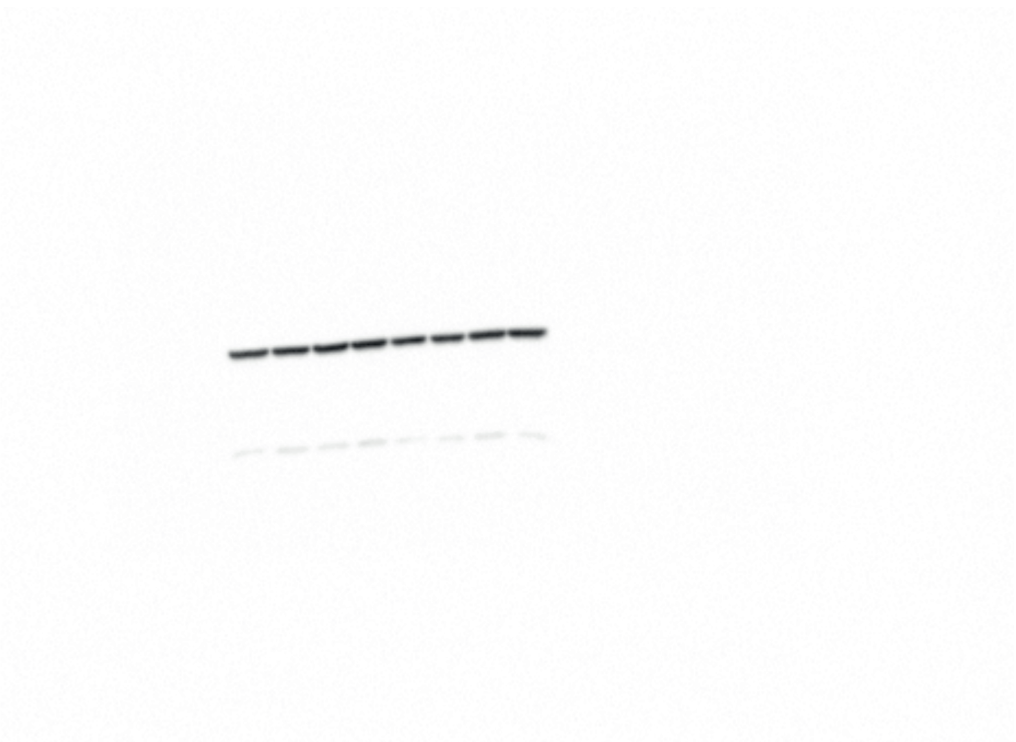

Chemiluminesence

250
150
100
75
50
37
25

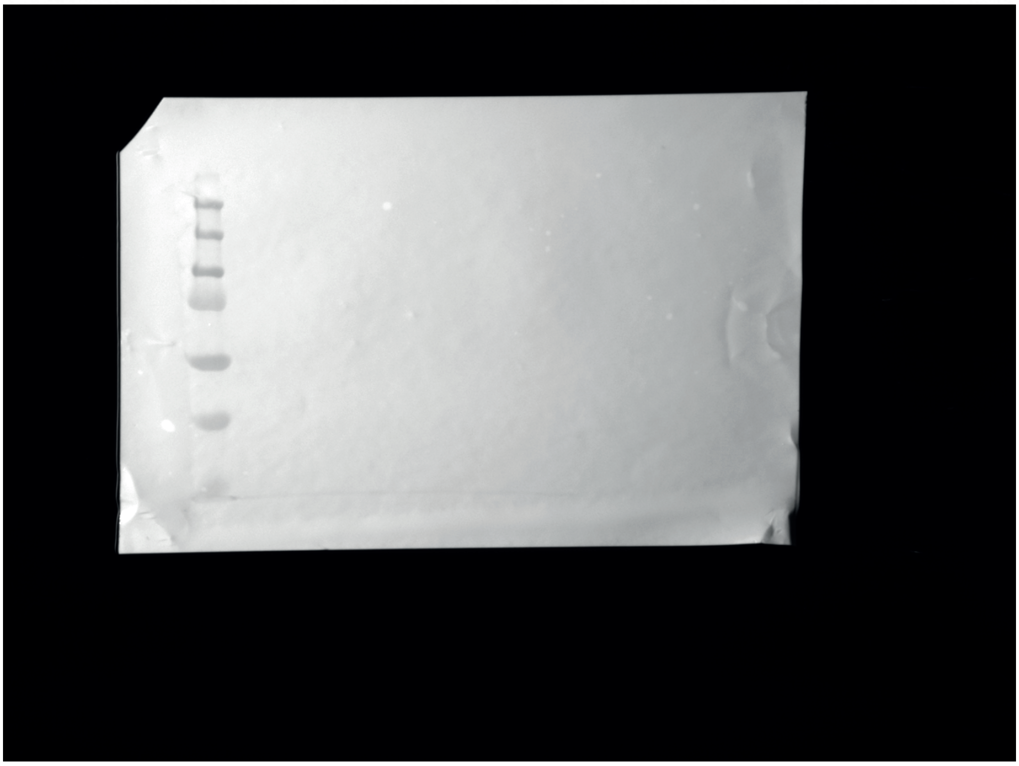

Photo

Tubulin

Fig S1A

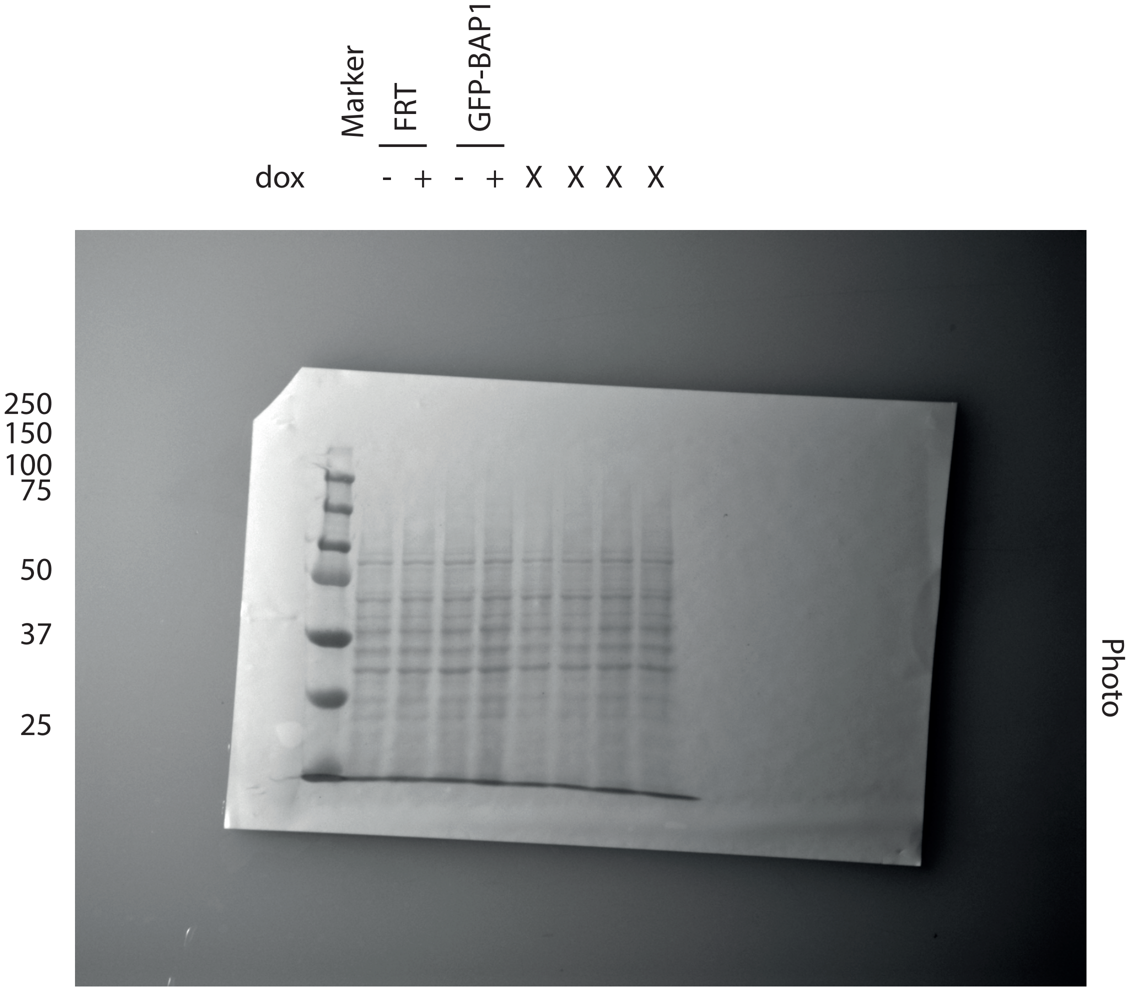

Ponceau

Fig S5

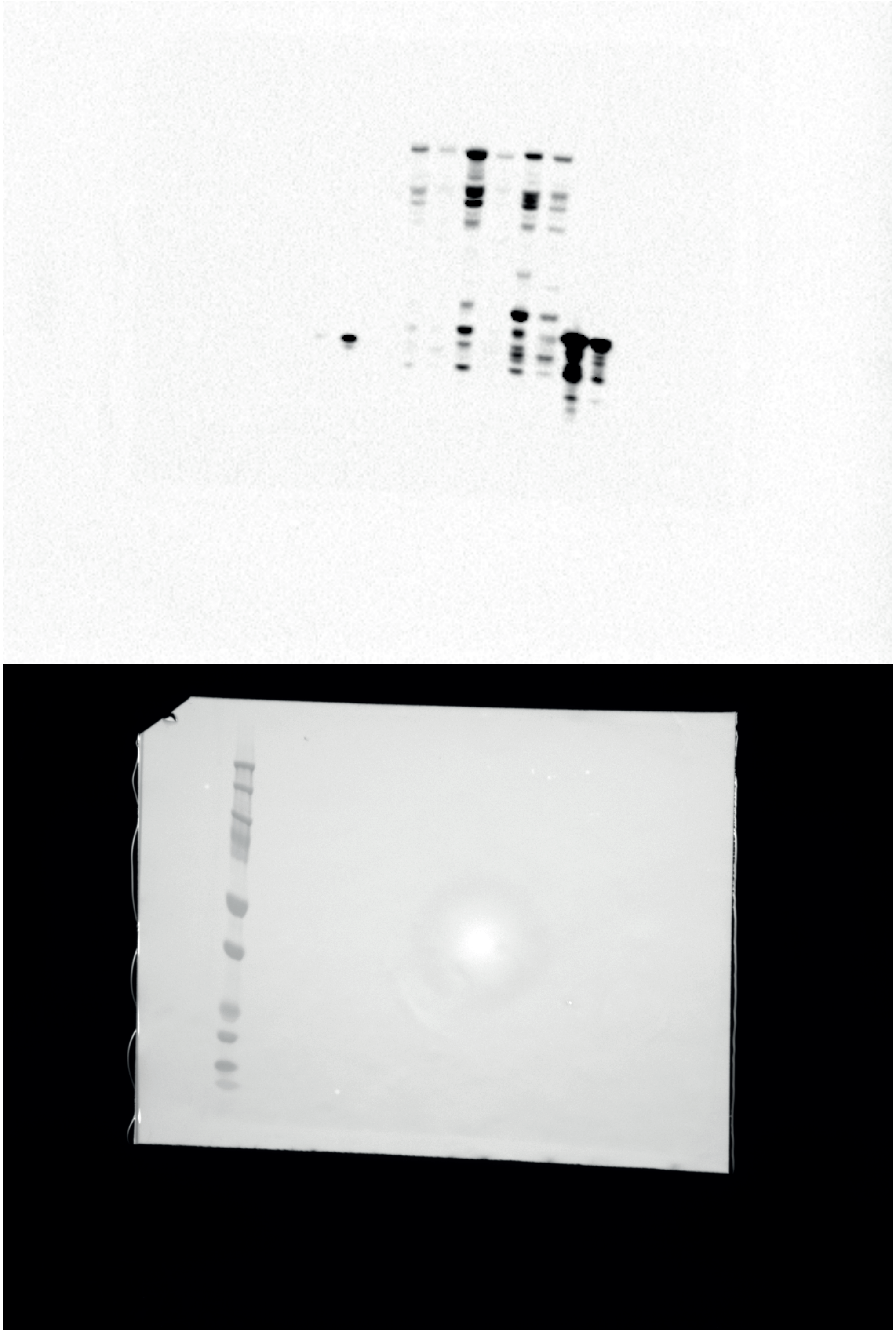
BAP1
Marker
X X X
Rec GFP
30
FL
2-670
Δ671-710
N-Stop
C N C N C N C N
Chemiluminesence
250
150
100
75
50
37
25
20
15
10
Photo
GFP

Fig S5

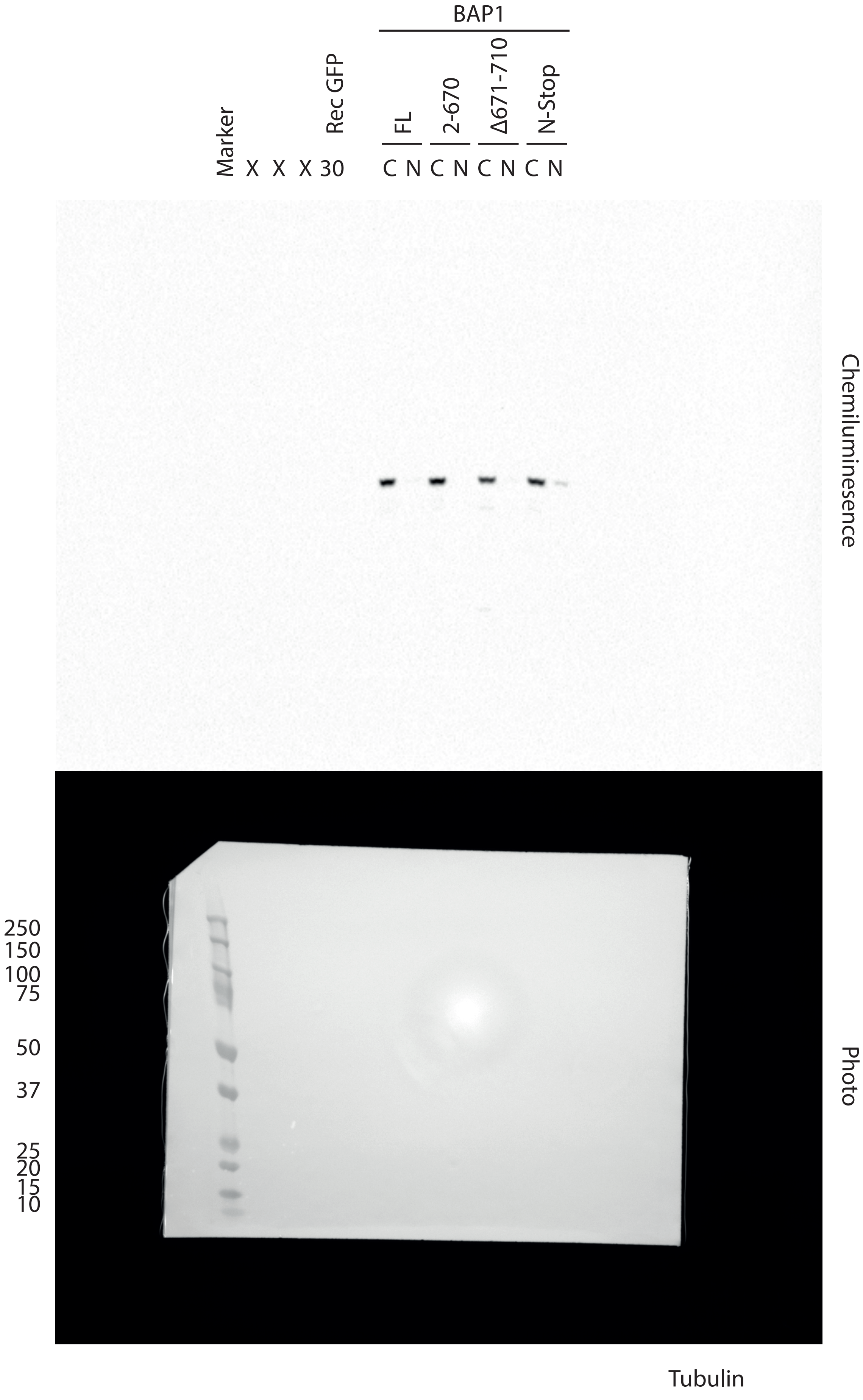
BAP1
Marker
X X X
Rec GFP
30
FL
2-670
Δ671-710
N-Stop
C N C N C N C N
Chemiluminesence
Photo
250
150
100
75
50
37
25
20
15
10
Tubulin

Fig S5

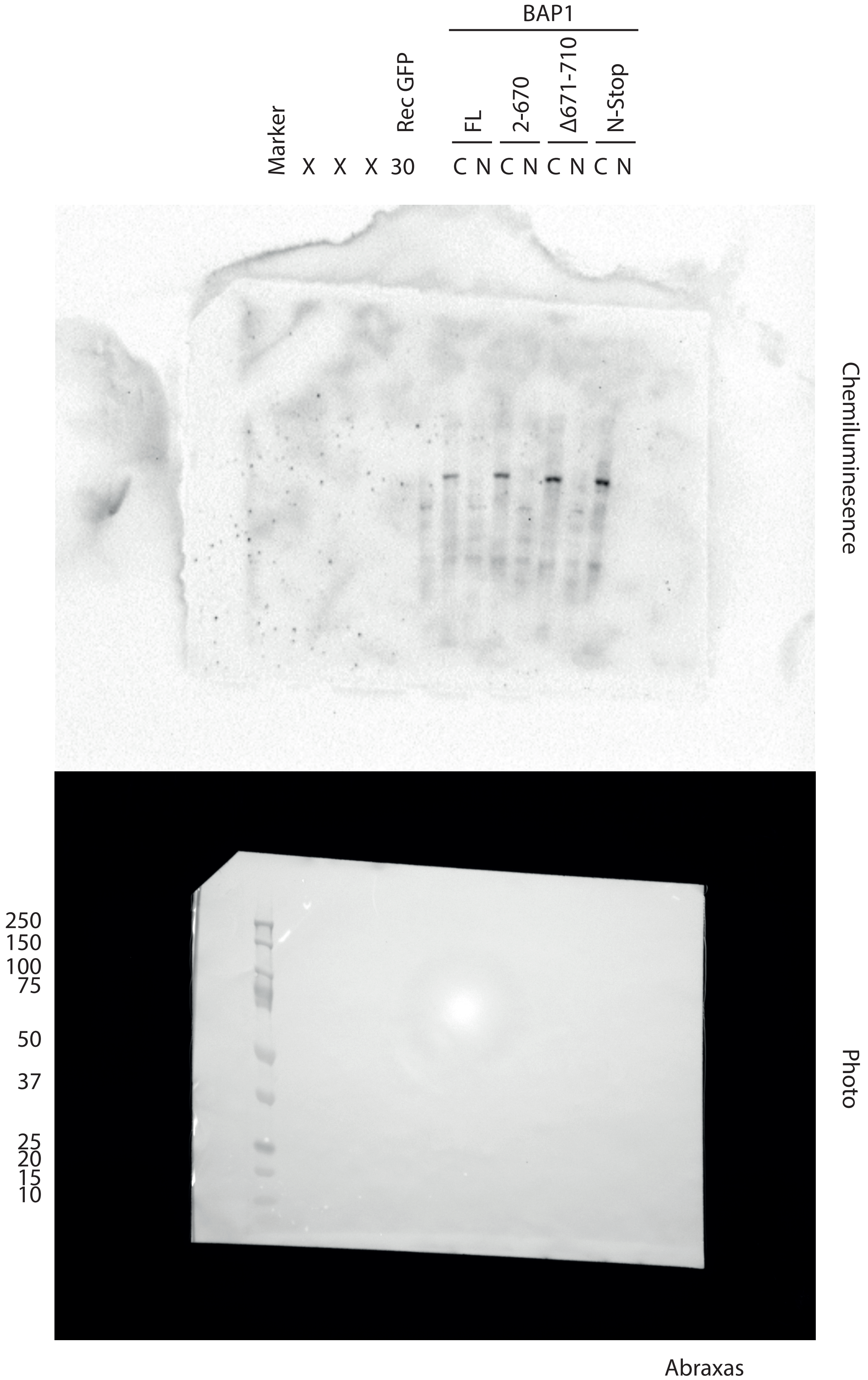
BAP1
Marker
X X X
Rec GFP
30
FL
2-670
Δ671-710
N-Stop
C N C N C N C N
Chemiluminesence
250
150
100
75
50
37
25
20
15
10
Photo
Abraxas

Fig S5

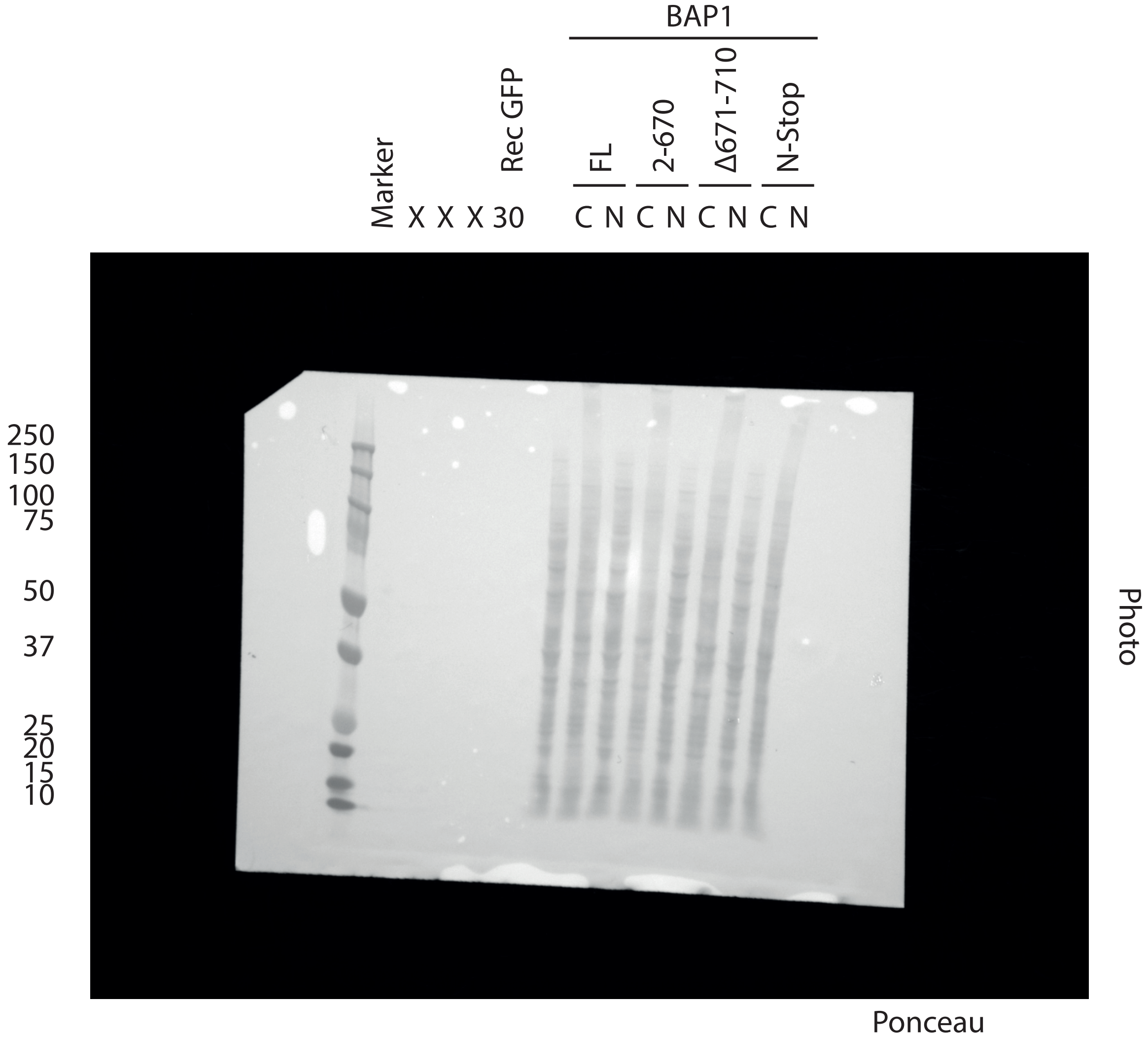
BAP1
Marker
X X X
Rec GFP
30
FL
2-670
Δ671-710
N-Stop
C N C N C N C N
250
150
100
75
50
37
25
20
15
10
Photo
Ponceau
