# Supplementary material for: Proteomic analysis identifies novel binding partners of BAP1
Source: PLoS One. 2021 Sep 30;16(9):e0257688. doi: 10.1371/journal.pone.0257688 (PMC8483321; doi:10.1371/journal.pone.0257688)
Supplement: Attachment — Submitted filename: 115169_1_rebuttal_2061359_qskmfr.docx [file pone.0257688.s010.docx]

**Reviewer response**

We thank the reviewers for their response. All reviewers find our study (potentially) interesting and/or a resource to gain further understanding on BAP1 molecular functions. They also have some common comments.

The reviewers would prefer to see further characterization of the interactions and their functional effects. We would have liked to address this but found for COPI that knockdown of these genes is lethal, whereas on the BAP1 side the interactions are mapped to the functionally critical C-terminus, making these experiments technically extremely challenging. These issues, unfortunately, preclude further validation studies at this point. Nevertheless, we do feel that the quality of our interaction dataset is such that it is be worth publishing these finding for this important tumor suppressor.

Most reviewers would like us to place the data more in context. To address this, we have extended the discussion, highlighting the essence of our findings and how we envisage this could impact BAP1 function.

Finally, both reviewer 1 and 3 would like the results section to be more succinct and we have shortened it to improve readability.

Other points are addressed in the point-by-point response to individual reviewers below.

**Point-by-point response to reviewers**

Reviewer #1 (Evidence, reproducibility and clarity (Required)):

Very interesting study on BAP1 tumor suppressor. The work needs further characterization of the interacting partners identified.

Reviewer #1 (Significance (Required)):

BAP1 is an important tumor suppressor mutated in several malignancies and the mechanism of action of this deubiquitinase are far from being completely understood.

This interesting work aimed at identifying novel cytoplasmic partners of BAP1 which can highly relevant to its tumor suppressor function. BAP1 is predominantly nuclear, but can also be found in the cytoplasm. New insights into the cytoplasmic functions of BAP1 are needed.

The manuscript is overall well written and the data are very solid.

The manuscript would need additional work before acceptance

**Comments**

Reviewer #1
1) The abstract can be improved to reflect the data of the manuscript.

Unfortunately, we do not understand what part of the abstract is meant by the reviewer, which makes it hard to address.

2) The result section, manuscript could emphasize the results rather that the technical aspects

We've improved readability of this part of the manuscript by moving technical parts that are not required for interpretation of the results to the material and method section, a supplementary text and a new supplemental figure 1 (causing the original numbering to shift).

3) It would be interesting to further investigate the significance of some key interactions

We agree that these questions are of importance (see reviewer 2 point 2, reviewer 3 point 1). We have tried to address these questions using gene knockdown techniques. However, the importance of regulation of protein transport and vesicle formation by COPI translates to lethal effects on cell viability upon knockdown of these genes making these experiments technically impossible to execute. Further functional investigation is technically and financially beyond the scope and possibilities of this paper.

4) The discussion is quite short and can put the findings in perspective

We've extended the discussion to place results into perspective, also regarding the potential role of BAP1 activity towards potential substrates (point 8). This will help to highlight the important findings of the research.

5) It would be interesting to test some cancer-associated mutations

The interaction is in the C-terminus of BAP1, which combines several functions[1, 2]. This would dramatically complicate the interpretation of results. Particularly the presence of the NLS, a major regulatory posttranslational modification[3] and the recruitment signal for nucleosomes could all interfere with BAP1 function independently.

6) Figure 4 can be improved

Thanks for this comment. We have increased readability of the figure by addition of schematic representations of the used constructs, a legend that explains the color-coding of the interactors. We have also removed dotted lines to make it less busy.

7) Yu et al MCB 2010 is one of the key papers on BAP1 purification and can be cited

We apologize for omitting this reference and have included it in the revised manuscript.

8) The authors can discuss potential substrates of BAP1 and mechanism of deubiquitination

We've extended the discussion to this extent.

**Referee Cross-commenting**

I agree with the comments of Reviewer #2


Reviewer #2 (Evidence, reproducibility and clarity (Required)):

**Summary:**

BAP1 is a deubiquitinase that mainly functions to control H2Aub levels in nucleus. BAP1 is a tumor suppressor with mutations or deletions in several human cancers. Previous studies have identified many interacting proteins with BAP1, most notably its binding partners involved in PR-DUB complex, such as ASXL1/2, FOXK1/2, HCFC1, OGT etc. In this study, by using FRT-mediated recombination, the authors generated tagged-BAP1 expressed from its endogenous promoter and conducted AP-MS analysis to identify BAP1 interacting proteins in both nuclear and cytoplasmic fractions. These analyses identified several new BAP1-interacting proteins in cytoplasmic fractions, including histone acetyltransferase 1 (HAT1) and the heptameric coat protein complex I (COPI, which is involved in protein sorting and trafficking). The authors further confirmed the interactions between BAP1 and HAT1 (as well as a COPI subunit) at the endogenous level.

**Major comments:**

Overall, the current study has relatively limited data with limited scopes: basically a proteomic study focusing on one single protein (which has already been subjected to several proteomic studies in previous publications). There is a significant room for authors to improve this potentially interesting study (see below for specific comments), although this may take substantial additional efforts.

1. In the current manuscript, there is no data to further characterize the interactions between BAP1 and HAT1 (or COPI). For protein-protein interaction studies, readers generally are interested in information such as whether these bindings are direct or indirect (particularly for COPI because it contains multiple subunits), and which regions mediate the interactions?

Our mass spectrometry data suggests binding of BAP1 to be mediated through its C-terminus. Mutations of the KxKxx domain have shown that this motif is not involved. Mapping the interaction any more specifically is likely to be very difficult as the C-terminus of BAP1 is involved in many different functions and contains many important elements (ULD domain, CTE, NLS) required for its function. Mutational analysis aimed to map the interaction will induce many secondary effects as the protein localization and substrate targeting will be severely affected as shown by us and other research groups. Identifying what subunit of the COPI complex is mediating the interaction requires purification of these proteins along with purified full length active BAP1, for which attempts have been made but were still unsuccessful. Further investigation is technically and financially beyond the scope and possibilities of this paper.

2. No data to study the functional significance of the identified protein-protein interactions. This is a major weakness of the current study. For example, HAT1 is a histone acetyltransferase and mainly functions in the nucleus. Does the BAP1-HAT1 interaction in cytoplasm suggest that they have functions in cytoplasm independent of their canonical function in regulating transcription in the nucleus? Likewise, does BAP1-COP1 interaction suggest that somehow BAP1 is involved in regulating protein sorting and trafficking?

Please see our general response and reviewer 1 point 3.

3. The identified interactions appear to be weak. These proteins are located near the edge of the significance curve in volcano plots (Fig. 1C-1D and others). The IP data also appear to be weak; for example, see Fig. 5D, it's hardly to see COPA blot in BAP1 IP. The COPA IB signal from 5% input WCL is probably hundred-fold stronger than that from BAP1 IP. Weak interactions do not necessarily mean they are not important; however, there is no functional data to support this claim (see the point above).

The essence of our paper is that the interactions which are barely visible in figure 1, gain significance in the absence of endogenous BAP1 to the point where all COPI subunits are as confidently identified as previously validated BAP1 interactors like ASXL, FOXK and HCFC proteins (figure 4). Our quantifications indicate that the new interactors have lower stoichiometry, and this may explain why they were harder to identify. This observation is discussed in the discussion section.

4. It seems that the entire study focuses on one specific cell line. Repeat the analyses in other cell lines can help boost the robustness and significance of the study.

As discussed under the previous point, the removal of endogenous BAP1 was important for significance, and since BAP1 is a common essential gene, we don't have any other cell lines in which this would be possible. However, we have been able to confirm the HAT1 interaction with BAP1 in U2OS on endogenous levels (Fig 1E,F).

The major interacting proteins they identified from the cytoplasmic fraction are still those mainly localized in nucleus (such as HCFC1, FOXK1/2). A western blotting to show nuclear vs cytoplasmic fraction is required.

Our immunoblot containing cytoplasmic and nuclear input samples used for figure 4 show proper separation of fractions without major leakage as shown in supplemental figure 4 (Tubulin and Abraxas lane as cytoplasmic and nuclear markers respectively), while iBAQ data show a substantial amount of protein to be bound (stoichiometry.xls). This is corroborating with the sample correlation data shown in supplemental figure 5B which shows very little correlation between cytoplasmic and nuclear samples in the mass spectrometry experiment. These data show that the interactions of the cytoplasmic partners that are mainly localized in the nucleus are real interaction and are not due to mixing of cellular compartments.

**Minor comments:**

6. page 9 "A GFP coIP experiment of both GFP-BAP1 and the catalytic-dead BAP1 C91S mutant shows coimmunoprecipitation of HAT1 (Figure 1F)." here Fig. 1F should be Fig. 1E.

The Figure was indeed mislabeled. Because of the addition of a new supplemental figure for reviewer 1 point 2 some figure numbers have shifted. The old figure 1E has now become 1C and is now numbered accordingly.

Reviewer #2 (Significance (Required)):

The current study is limited in scope. Without functional data for these interactions, the overall significance of the study is likely limited.

**Referee Cross-commenting**

My overall assessment is similar to the other two reviewers (particularly reviewer 3): the study is rather descriptive, limited in scope, and lacks mechanistic understanding of BAP1 functions: for example, see reviewer 1 comment "it would be interesting to further investigate the significance of some key interactions"; reviewer 3 comment "The present manuscript contained very little information beyond description of BAP1 interactomes and subsequent validation of BAP1-COPI interaction. In the very least, I would recommend for the authors to explore contextual significances and/or regulations of the novel BAP1-COP1 interaction."


Reviewer #3 (Evidence, reproducibility and clarity (Required)):

In the present manuscript, Baas and colleagues seek to identify novel BRCA1-associated protein 1 (BAP1) interacting partners. To do so, the authors performed affinity purifications of different GFP-tagged BAP1 constructs in combination with mass spectrometry from either wild-type HeLa or those, which endogenous BAP1 expression had been knocked-out using CRISPR/Cas9. MS analysis of the pull-downs revealed COPI as a novel cytoplasmic interactor for the full-length GFP-BAP1 in addition to to other previously known BAP1 interactors such as HAT1, ASXL1/2, FOXK1/K2, OGT.

The authors subsequently went on to validate the BAP1-COPI interaction and observed that such interaction was independent of the canonical COPI binding motifs KxKxx present in the BAP1 C-terminus.

**Major comments:**

The present manuscript contained very little information beyond description of BAP1 interactomes and subsequent validation of BAP1-COPI interaction. In the very least, I would recommend for the authors to explore contextual significances and/or regulations of the novel BAP1-COP1 interaction.

Please see our general response and reviewer 1 point 3.

The present manuscript could be written in more concise manner.

We have shortened the results section as discussed under reviewer 1 point 2 to make it more concise.

Reviewer #3 (Significance (Required)):

While the present study may provide a resource to gain further understanding on BAP1 molecular functions, it is very difficult to appreciate the significance of the presence manuscript in the current descriptive form.

We have expanded the discussion to better explain the significance of our findings.

1.	Sahtoe, D.D., et al., *BAP1/ASXL1 recruitment and activation for H2A deubiquitination.* Nat Commun, 2016. **7**: p. 10292.

2.	Ventii, K.H., et al., *BRCA1-associated protein-1 is a tumor suppressor that requires deubiquitinating activity and nuclear localization.* Cancer Res, 2008. **68**(17): p. 6953-62.

3.	Mashtalir, N., et al., *Autodeubiquitination protects the tumor suppressor BAP1 from cytoplasmic sequestration mediated by the atypical ubiquitin ligase UBE2O.* Mol Cell, 2014. **54**(3): p. 392-406.
